# Targeting Mitochondria in Aging‐Related Diseases: Therapeutic Potential and Obstacles

**DOI:** 10.1002/mco2.70790

**Published:** 2026-06-04

**Authors:** Zijie Xiang, Yu Chen, Xishui Liu, Haowen Lu, Yuqing Yang, Lei Xing, Yu Zhang, Chuandong Lang, Siming Zhang, Shixiang Zhao, Youzhi Hong, Jiaxiang Bai, Yusen Qiao

**Affiliations:** ^1^ Department of Orthopedics The First Affiliated Hospital of Soochow University Suzhou Jiangsu China; ^2^ Department of Breast Surgery Changhai Hospital Naval Medical University Shanghai China; ^3^ Department of Orthopedics The First Affiliated Hospital of USTC University of Science and Technology of China (USTC) Hefei Anhui China; ^4^ Department of Hematology The First People's Hospital of Yunnan Province Kunming China

**Keywords:** aging, mitochondria, therapy

## Abstract

Aging is a complex biological process characterized by the functional decline of multiple cellular organelles, with mitochondrial dysfunction emerging as a predominant hallmark. Alterations in mitochondria within senescent cells primarily encompass two interrelated aspects: intrinsic mitochondrial dysfunction and compromised mitochondrial quality control systems, including mitophagy, dynamics, and biogenesis. However, a comprehensive synthesis that bridges mechanistic insights into mitochondrial dysfunction with an analysis of therapeutic obstacles remains lacking. Here, we systematically summarized the pathways leading to mitochondrial dysfunction in aging and deeply analyzed how this dysregulation, including mitochondrial DNA instability and mitochondria driving inflammation through the cGAS–STING pathway, contributed to the etiology of aging‐related diseases, including muscle, bone, neurodegeneration, cardiovascular, and metabolic diseases. Additionally, we analyzed a series of mitochondrial targeted treatment strategies, from metabolism and kinetic regulation to disease‐specific intervention and emerging technologies, such as mitochondrial transplantation and mitochondrial DNA base editing. Finally, we emphasized the key obstacles that must be overcome for clinical transformation, including tissue‐specific mitochondrial heterogeneity. By combining the basic mechanism with the development of treatment and its potential challenges, this review provides a key perspective for promoting the emerging field of mitochondrial medicine to intervene in aging‐related pathology more accurately and effectively.

## Graphical abstract

This article systematically summarized the specific mechanism of aging‐related diseases caused by mitochondrial dysfunction, and summarized the broad‐spectrum treatment methods and disease targeted treatment strategies for mitochondria. At the same time, it also pointed out the dilemma faced by mitochondrial targeted treatment. Finally, we made a prospect for the future development of this emerging field.

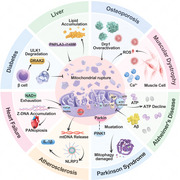

## 1 Introduction

The global increase in human life expectancy results in ongoing population aging, imposing a growing socioeconomic and healthcare burden due to age‐related chronic conditions. This phenomenon is especially notable in China, where the percentage of individuals aged 65 years and older is anticipated to double from 12% in 2019 to 26% by 2050, thereby introducing unparalleled socioeconomic obstacles [1, 2]. Organismal aging is a multifaceted, gradual process marked by the step‐by‐step decline of cellular and systemic functions, as well as the deterioration of various tissues, resulting in compromised function and heightened mortality risk [3]. In the 1920s, Raymond Pearl noted a negative association between metabolic rate and lifespan while investigating aging mechanisms [4]. Expanding on this observation, Denham Harman introduced the free radical theory of aging in the 1950s, suggesting that reactive oxygen species (ROS) could stem from mitochondria, causing the buildup of impaired proteins, lipids, and DNA. This process, he argued, contributes to age‐related diseases through an inevitable yet random mechanism [5]. Following this, the connection between mitochondrial biology and aging has become a focal topic for many researchers [6].

Mitochondria, as energy providers, are closely associated with age‐related diseases. Mitochondrial dysfunction is widely recognized as one of the core hallmarks of aging and its associated pathologies. The defining characteristics of mitochondrial dysfunction in aged tissues and cells include diminished respiratory capacity and reduced mitochondrial membrane potential (MMP) [3]. Mitochondrial dysfunction at the cellular level is a fundamental contributor to aging, resulting in significant aging characteristics such as DNA damage, epigenetic alterations [7], the emergence of the senescence‐associated secretory phenotype, and compromised intercellular signaling [8]. Therefore, the maintenance of mitochondrial functional competence is crucial for preserving cellular equilibrium, regulating metabolism, and ensuring viability.

Mitochondria undergo significant structural and functional decline during aging, primarily manifested as: accumulation of mitochondrial DNA (mtDNA) mutations [9], ROS production [10], decreased ATP synthesis efficiency, and impaired mitochondrial autophagy [11]. These dysfunctions do not occur in isolation but form a vicious cycle, mutually reinforcing each other: mtDNA damage leads to defects in respiratory chain complexes, increasing ROS leakage; excessive ROS further damages mtDNA, lipids, and proteins while suppressing mitochondrial autophagy, lead to the buildup of dysfunctional mitochondria. Ultimately, this triggers apoptosis, inflammatory responses, and stem cell depletion, directly driving tissue functional decline and the development and progression of age‐related illnesses [12]. Moreover, recent research has unveiled the role of mitochondrial stress in initiating a proinflammatory state, termed inflammaging, through the release of mtDNA fragments that act as damage‐associated molecular patterns (DAMPs), further exacerbating tissue dysfunction. Current research predominantly focuses on studying these mechanisms independently, creating a significant knowledge void regarding the reciprocal communication and cooperative relationships of mitochondria in the aging processes at the cellular and organismal levels.

Research indicates that aging and age‐related diseases are closely associated with an imbalance between energy supply and demand. This imbalance may be mitigated through various interventions, such as physical activity, caloric restriction, and the activation of natural molecular pathways that target conserved longevity mechanisms [13]. Given the central role of mitochondria in age‐related diseases, targeting mitochondria has emerged as a notably auspicious therapeutic approach for delaying the aging process and preventing/treating multiple age‐related disorders. Current research directions primarily encompass: developing mitochondria targeted antioxidants for precise clearance of mitochondrial ROS [14], activating mitochondrial autophagy to eliminate damaged mitochondria [15], supplementing mitochondrial cofactors [16, 17], enhancing mitochondrial biogenesis [18], and improving mitochondrial dynamics [19]. Beyond these, cutting‐edge approaches like mitochondrial transplantation and gene editing tools targeting mtDNA are emerging as revolutionary strategies with the potential to restore mitochondrial integrity. Certain strategies have demonstrated promise in enhancing mitochondrial function and delaying disease advancement in preclinical models and even early clinical trials [20].

However, translating mitochondrial targeted therapies into safe and effective clinical interventions remains fraught with significant challenges: how to enable drugs to efficiently and specifically penetrate the double mitochondrial membrane to reach their target sites [21]; cellular and tissue heterogeneity necessitates therapies that adapt to variations in mitochondrial function across different tissues [22]. This heterogeneity is recently being mapped at an unprecedented scale, with studies revealing tissue‐specific “mitochondrial molecular clocks” based on mutational patterns, underscoring the need for personalized chronological assessments. Potential off target effects and long‐term safety require careful evaluation; complex diseases necessitate consideration of multipathway synergistic effects; and substantial interindividual variations in genetic background and mitochondrial function demand personalized therapeutic strategies [23]. Furthermore, the clinical translation of certain drugs effective in aging animal models remains under intensive investigation [24].

This review systematically elucidates the alterations in mitochondrial function associated with aging, emphasizing the effects of mitochondrial homeostasis imbalance and mtDNA release on senescent cells. Simultaneously, we examine the fundamental mechanisms of mitochondrial dysfunction in age‐related diseases that impact the skeletal and cardiovascular systems, as well as neurodegenerative and metabolic disorders. We will critically assess current therapeutic strategies aimed at enhancing mitochondrial function or mitigating damage, along with the mechanisms that underpin these approaches. These approaches will be analyzed in detail under two broad categories: general therapeutic strategies and disease‐specific targeting strategies. We will examine their specific mechanisms and the key technical barriers encountered during translation to clinical applications. By integrating the latest molecular mechanisms, this review provides a comprehensive guide for understanding the mitochondrial basis of aging and advancing the development of antiaging therapies.

## 2 Age‐Related Alterations in Mitochondria

Mitochondria are indispensable organelles central to energy metabolism and intracellular signaling regulation. Mitochondrial dysfunction can significantly disrupt cellular energy conversion and lead to abnormal alterations in signaling pathways, ultimately jeopardizing cell function. Cellular aging is a multifaceted process marked by a progressive decline in organelle function, with mitochondrial dysfunction serving as a key hallmark [25].

### 2.1 Mitochondrial Quality Control in Aging

Cellular mitochondria are governed by mitochondrial quality control mechanisms that are crucial for sustaining normal function. With advancing age, processes such as mitophagy, mitochondrial dynamics, and the mitochondrial unfolded protein response are markedly diminished. Importantly, changes in mitochondrial quality control frequently occur prior to the onset of overt cellular senescence. We propose that the analysis of mitochondrial quality control signals can reveal significant targets and clarify the roles of different quality control mechanisms in restoring the function of senescent cells, thus offering viable strategies for the treatment of aging‐related diseases.

#### 2.1.1 Mitophagy in Aging

To maintain mitochondrial quality and eliminate damaged or superfluous organelles, mitochondria have evolved various protective mechanisms. The removal of whole mitochondria happens via a specific type of autophagy known as mitophagy [26]. Numerous studies indicate that mitophagy declines with age, promoting the buildup of impaired mitochondria, linked to several disorders such as heart disease [27], myocardial hypertrophy [28], neurodegenerative diseases [29], cancer [30], and normal aging [31].

Given that the molecular mechanisms of mitophagy is crucial for targeting it through pharmacological interventions. In mammals, mitophagy receptors broadly initiate the process in either a Ub‐dependent or Ub‐independent manner [32].

Ubiquitin‐mediated mitophagy primarily involves the PINK1/Parkin signaling pathway. PINK1, a serine/threonine‐protein kinase, accumulates on impaired mitochondria upon depolarization, undergoes autophosphorylation [33], and recruits and activates the E3 ubiquitin ligase Parkin [34, 35]. Activated Parkin ubiquitinates mitochondrial outer membrane proteins, marking the organelle for autophagic degradation. This pathway is primarily activated under mitochondrial stress, allowing healthy mitochondria to escape PINK1/Parkin‐mediated removal [36].

Another class of ubiquitin‐independent mitophagy receptors lacks a UBD but possesses a concise linear LC3‐interacting region motif [26, 37]. This motif enables receptors to engage with LC3/GABARAP proteins on the phagophore, thereby facilitating the autophagic degradation of specific cargo [37].

#### 2.1.2 Alterations in Mitochondrial Dynamics in Aging

Mitochondria maintain a functional state through continuous fusion and fission. Dynamin‐related proteins including mitofusin 1/2 (MFN1/2) [38], optic atrophy 1 (OPA1) [39], and dynamin‐related protein 1 (Drp1) [40] collectively oversee mitochondrial dynamics [41]. Specifically, MFN1/2 facilitate the fusion of the outer mitochondrial membrane, OPA1 is responsible for the fusion of the inner mitochondrial membrane, and Drp1, which is predominantly expressed in the cytoplasm, mediates mitochondrial fission [42, 43, 44].

Research on loss‐of‐function and overexpression reveals that mitochondrial dynamics play a crucial role in regulating metabolism in various cells and tissues [42]. For example, depletion of MFN2 results in mitochondrial dysfunction, including decreased oxygen consumption linked to ATP synthesis [45], heightened proton leak, reduced MMP, increased ROS production [46], and lowered coenzyme Q (CoQ) levels [47]. These alterations impair the oxidation of glucose, pyruvate, and fatty acids [48]. OPA1, whose cleavage is dependent on MFN2, is linked to changes alterations in mitochondrial metabolism and is implicated in the regulation of cristae morphology, respiratory efficiency, and the formation of respiratory chain supercomplexes in mice [49].

Drp1 is associated with alterations in mitochondrial function. For instance, prolonged in vitro passaging of bone marrow mesenchymal stem cells (BMSCs) leads to an imbalance in mitochondrial dynamics, notably causing disturbances in mitochondrial structure and function, which play a crucial role in driving replicative senescence. Increased passages lead to significantly reduced translocation of Drp1 from the cytoplasm to mitochondria, resulting in impaired fission, abnormal morphology, ROS accumulation, and dysregulated energy metabolism, subsequently triggering increased expression of senescence markers like p21, diminished proliferation and differentiation capacity, and loss of therapeutic function. Notably, small extracellular vesicles from young stem cells from human exfoliated deciduous teeth can promote Drp1 translocation to mitochondria, remodeling mitochondrial dynamics balance, reverse dysfunction, and effectively ameliorate the replicative senescence phenotype in BMSCs, restoring their stemness, immunomodulatory capacity, and in vivo therapeutic efficacy [50]. Available data indicate that maintaining a precise equilibrium between fusion and fission is essential for sustaining mitochondrial metabolism.

Mitochondrial dynamics, being pivotal for cellular homeostasis, are linked to a spectrum of diseases through mutations or modifications in fusion and fission proteins. The disruption of these dynamics significantly contributes to the pathogenesis of numerous age‐related disorders [42].

#### 2.1.3 Mitochondrial Unfolded Protein Response in Aging

Disruptions in proteostasis and compromised mitochondrial metabolic function can hinder mitochondrial protein import, leading to the initiation of the mitochondrial unfolded protein response under stress (UPRmt) [41, 51]. UPRmt, a mitochondrial stress response, is evident observed in both C. elegans and mammalian systems [52, 53]. Recognized activators comprise impaired electron transport chain (ETC) function, modified mitochondrial dynamics, accumulation of unfolded proteins, mtDNA depletion, and inhibition of mitochondrial chaperones or proteases, alongside elevated ROS levels [54, 55, 56, 57, 58]. Mitochondrial damage triggers UPRmt, which prompts the elevation of proteases, chaperones, and other stress response genes to boost cellular proteostasis and alleviate mitochondrial damage [59].

The UPRmt is regulated by multiple targets. For instance, the transcription factors CHOP and ATF4 can directly stimulate the transcriptional expression of ATF5 [60]. ATF5 is typically localized in mitochondria under normal conditions. Elevated levels of ROS or stress in mitochondrial protein folding lead to the accumulation of ATF5 in the cytosol. Subsequently, it is translocated to the nucleus, where it governs the transcription of mitochondrial chaperones and proteases, thereby initiating UPRmt during mitochondrial stress [52]. While ATF5 does not impact mtDNA replication, it boosts the transcription of various chaperones and proteases to aid in the recovery from mitochondrial stress [61].

Sirtuin 1 (SIRT1), a histone deacetylase involved in fatty acid synthesis, oxidation, and adipogenesis, can significantly reduce oxidative stress and ROS production and alleviate mitochondrial dysfunction upon activation [62, 63, 64]. Research has demonstrated that increasing SIRT1 suppresses the PERK–eIF2α–CHOP pathway, leading to a decrease in endoplasmic reticulum stress, safeguarding chondrocytes against apoptosis, and improving the advancement of osteoarthritis in rat models. Investigating this pathway further could provide novel perspectives for osteoarthritis investigations [65].

Meanwhile, mitochondrial Lon protease (LonP1) is a crucial regulatory hub within the organelle [66]. It binds mtDNA in a sequence‐specific manner and plays a vital role in mtDNA maintenance [67]. Even mild LonP1 deficiency is associated with cell death [68]. Researchers using a Drosophila model with inactivated Lon protease found that loss of LonP1 function led to the accumulation of mitochondrial unfolded proteins, triggering UPRmt and significantly inhibiting the translation of mitochondrially encoded proteins. This translation deficiency resulted in respiratory chain dysfunction, reduced ATP synthesis, decreased motor ability, and shortened lifespan. The partial alleviation of the phenotype was demonstrated by the overexpression of another matrix protease, ClpP, confirming that the core pathogenic factor is the accumulation of unfolded proteins [69]. This study was the pioneering work to unveil that LonP1 inactivation induces aging‐related degenerative alterations via UPRmt‐mediated translational inhibition.

In the UPRmt pathway, SIRT1, as an upstream regulator, can promote ATF5‐mediated mitochondrial stress response by activating the expression or enhancing its activity, help cells cope with mitochondrial dysfunction, and jointly maintain mitochondrial protein homeostasis and cell health [70]. Their interaction is of great significance in the pathological process of aging, muscle atrophy, neurodegenerative diseases, and other diseases, and is an important target for the treatment of related diseases. On the other hand, UPRmt promotes signal transmission between mitochondria and the endoplasmic reticulum by activating proteins such as LonP1 and SIRT1, enhances the communication function of mitochondrial endoplasmic reticulum contact sites (MERCs), maintains mitochondrial integrity, reduces oxidative damage, and helps maintain heart health and function [71]. In acute myocardial infarction, LonP1 decreases and activates UPRmt through eIF2α–ATF5 pathway to promote mitochondrial repair [72]. In dilated cardiomyopathy, the reduction of SIRT1‐3 weakens the UPRmt response. SIRT1 and LonP1 are not only encoded in the nucleus, but then translated and inserted between MERCs as anchor proteins, thus promoting mitochondrial repair and functional recovery [71]. It can be seen that these molecules are dynamic and inseparable in maintaining mitochondrial homeostasis.

Research also indicates that UPRmt activation can promote the spread of harmful forms of mtDNA, such as mtDNA, which disrupts oxidative phosphorylation and impairs cellular function [52]. This evidence suggests that further activating UPRmt during stressful conditions could worse mitochondrial dysfunction and have a negative impact on functional recovery. Nevertheless, the crucial role of UPRmt in maintaining mitochondrial homeostasis is well established.

#### 2.1.4 Mitochondrial Biogenesis in Aging

Regulation of organelle abundance and quality control involves mitochondrial biogenesis and mitophagy, which are opposing functions [73]. Mitochondria are not produced de novo but rather through a series of fusion and fission processes from existing organelles. Mitochondrial biogenesis includes mtDNA replication, transcription and translation of mtDNA‐encoded genes, and the incorporation of phospholipids and nuclear‐encoded proteins into different mitochondrial sub‐compartments [74]. Mitochondrial dysfunction can induce cell death and is characteristic of several human diseases such as diabetes, neurodegenerative disorders, and age‐related conditions [75].

Mitochondrial biogenesis relies on both mitochondrial and nuclear factors’ activity [73]. The peroxisome proliferator‐activated receptor γ coactivator‐1 (PGC‐1) family, including PGC‐1α, PGC‐1β, and PRC, regulates biogenesis and respiratory function [73]. PGC‐1α, the first discovered member, is a key regulator of mitochondrial biogenesis and function, orchestrating precise transcriptional cascades [76, 77]. In the skeletal system, the attenuation of this process directly leads to metabolic decline in osteoblasts and chondrocytes, accelerating the progression of osteoporosis and osteoarthritis [78, 79]. Recent research indicates that the Necdin protein family member promotes neuronal mitochondrial biogenesis by stabilizing PGC‐1α, inhibiting its degradation through the ubiquitin–proteasome system [76].

### 2.2 Shared Characteristics of Mitochondria in Aging‐related Diseases

Mitochondrial dysfunction, as a central component of energy metabolism and regulation of intracellular signaling, disrupts cellular metabolism and induces abnormal alterations in signaling pathways. This disruption ultimately contributes to the development and progression of aging and its associated diseases.

#### 2.2.1 mtDNA Instability

A primary mechanism of mitochondrial dysfunction is the instability of mtDNA. mtDNA lacks histone protection and has limited DNA repair capacity, making it highly vulnerable to attack by ROS generated within mitochondria during aging [80]. This leads to the accumulation of mtDNA point mutations, deletions, and depletion, which are associated with aging and age‐related diseases [81]. Such genetic damage directly impairs the synthesis of proteins related to the mitochondrial respiratory chain complexes, resulting in decreased oxidative phosphorylation efficiency and energy deficiency [82]. Furthermore, recent research has revealed a new role for free mtDNA fragments released from damaged mitochondria during aging: they act as DAMPs, activating the cytosolic cGAS–STING innate immune pathway, thereby triggering chronic, low‐grade inflammation, particularly prominent in neurodegenerative diseases and cardiovascular aging [83].

#### 2.2.2 ROS Imbalance

Imbalanced ROS generation and oxidative stress represent another crucial facet of mitochondrial dysfunction, forming a vicious cycle with mtDNA damage. According to the oxidative stress theory of aging, the primary driver of aging is oxidative damage induced by ROS [84]. Although ROS play a vital role as signaling molecules at normal levels, the aging process results in heightened leakage from the mitochondrial ETC and a reduction in the efficacy of intrinsic antioxidant mechanisms. This imbalance causes excessive ROS production that exceeds the cell's clearance capacity, leading to widespread oxidative damage. ROS can directly oxidize and modify protein components of the ETC complexes, further impairing their function [85]. Moreover, lipids, particularly cardiolipin unique to the inner mitochondrial membrane, are highly susceptible to peroxidation. Oxidized cardiolipin disrupts the MMP, impairs cristae structure, and promotes the release of proapoptotic factors from the intermembrane space, directly triggering apoptotic pathways, which is particularly detrimental in aging hearts and neurons [86].

#### 2.2.3 Calcium Homeostasis Dysregulation

Dysregulation of mitochondrial calcium homeostasis constitutes another important mechanism. Studies have identified two calcium channels, inositol 1,4,5‐trisphosphate receptor type 2 and the mitochondrial calcium uniporter, as senescence regulators in loss‐of‐function genetic screens [87]. ITPR2 triggers calcium release from the endoplasmic reticulum, and mitochondria uptake Ca^2^^+^ signals from the cytoplasm through the MCU, which is crucial for activating metabolic enzymes and regulating cell death. However, aging leads to dysregulation of the MCU complex, causing mitochondrial calcium accumulation.

The mitochondrial permeability transition pore (mPTP) is a protein complex in the inner membrane that can form a nonselective channel. This channel is voltage‐gated and activated by matrix calcium overload and ROS, with additional control from numerous associated proteins and posttranslational modifications of these proteins and the channel itself [88]. Excessive Ca^2^^+^ can induce abnormal prolonged opening of the mPTP. The complete opening of the mPTP results in the collapse of the MMP, interruption of ATP synthesis, burst production of ROS, and ultimately the release of proapoptotic factors and cell death [88]. This calcium dysregulation is particularly harmful in cells with high energy demands and complex calcium signaling, representing an important pathogenic mechanism in Parkinson's disease (PD) and heart failure (HF).

#### 2.2.4 Mitochondria‐Mediated Metabolic Abnormalities

With aging, the regulation of energy storage and consumption essential for cellular homeostasis becomes progressively disrupted, leading to notable metabolic abnormalities [25]. Typically, this metabolic dysregulation hastens the aging process, while interventions targeting metabolism have demonstrated the ability to prolong lifespan, highlighting the close link between aging and metabolism [89]. Key metabolites affecting mitochondrial function and consequently aging encompass those involved in the tricarboxylic acid (TCA) cycle and nicotinamide adenine dinucleotide (NAD^+^) [90, 91].

Growing evidence indicates that epigenetic modifications are a key hallmark of cellular aging. Histone posttranslational modifications, like methylation, acetylation, and ubiquitination, are pivotal in this phenomenon [92]. Metabolites generated in the mitochondrial TCA cycle, including pyruvate, α‐ketoglutarate (α‐KG), acetyl‐CoA, succinyl‐CoA, and fumarate, can instigate epigenetic changes in the nucleus via nonmetabolic routes [93]. These mitochondrial metabolites interact with the epigenome, inducing modifications in nuclear gene expression that effect cellular equilibrium and aging processes [25]. We focus here on two key metabolites: α‐KG and acetyl‐CoA.

α‐KG is a key TCA cycle metabolite and indispensable for cellular energy production and protein synthesis. As aging progresses, mitochondrial function progressively declines, leading to resulting in diminished mitochondrial metabolic flux and subsequently worsening α‐KG deficiency [94]. Meanwhile, the activity of α‐KG dehydrogenase complex, a key enzyme in the TCA cycle, is the lowest among them. During aging, the E2 subunit of this enzyme is inactivated due to thiol oxidation, leading to reduced TCA cycle flux, insufficient ATP generation, impaired signal transduction, and ultimately accelerated cellular senescence.

Studies indicate that as human follicular fluid ages, α‐KG levels tend to decrease significantly, and supplementing α‐KG can rejuvenate ovarian function in older mice [95]. Additionally, α‐KG has been demonstrated to alleviate age‐related declines in fertility among mammals [96]. In a study on lifespan, researchers found that α‐KG significantly extended C. elegans lifespan by approximately 50% by inhibiting ATP synthase, reducing ATP levels, activating autophagy, and inhibiting the TOR signaling pathway. α‐KG increases endogenously during nutrient scarcity and cannot further extend the lifespan of dietary restriction models or TOR‐deficient animals, indicating it mediates longevity by mimicking a dietary restriction state [97]. This mechanism is conserved in mammalian cells, providing a new target for aging intervention. This implies that adding α‐KG may prolong lifespan in different model organisms. In fruit flies, α‐KG extended lifespan through AMPK pathway activation and mTOR pathway inhibition [98]. Apart from its anti‐inflammatory and epigenetic impacts, α‐KG can function as an antioxidant and control nitrogen and ammonia balance. In humans, α‐KG exhibits therapeutic promise in heart, brain, liver, and skeletal muscle conditions and could impact human aging [99].

Acetyl‐CoA serves as a primary substrate in the TCA cycle, a pivotal hub in carbon metabolism, essential for fueling the respiratory chain and various biosynthetic pathways [25, 100]. It plays a fundamental role in cellular metabolism as a direct participant in the TCA cycle and critical acetylation reactions [101]. Throughout the aging process, the levels and intracellular distribution of acetyl‐CoA undergo dynamic alterations in response to mitochondrial disruptions or pathological conditions [102]. Simultaneously, age‐related modifications are observed in histone acetylation profiles across diverse tissues and organisms [25, 103]. A study of the human lateral temporal lobe found that histone H4K16ac acetylation modification globally increased during normal aging but was significantly reduced in Alzheimer's disease (AD) patients, with the age‐related gain in H4K16ac significantly negatively correlated with AD‐related loss [104]. Similarly, aged mice carrying the ApoE4 allele, a major genetic risk factor for AD, showed significant accumulation of senescent cells accompanied by significantly reduced acetyl‐CoA levels. Conversely, a systemic increase in hippocampal acetyl‐CoA levels reduced the number of senescent cells in the brains of ApoE4 aged mice, improved hippocampal synaptic plasticity, and delayed the onset of spatial cognitive impairment [105]. These findings suggest that metabolites originating from mitochondria play a role in mediating epigenetic histone modifications like acetylation, succinylation, and methylation. This involvement contributes to the control of gene expression as cells age, highlighting the crucial significance of mitochondrial TCA cycle metabolites in governing cellular senescence.

A noteworthy aspect of aging is the substantial reduction in levels of NAD^+^. NAD^+^ plays a crucial role as an electron acceptor in diverse cellular metabolic pathways, acting as a coenzyme for mitochondrial TCA cycle dehydrogenases and the ETC. Through metabolic processes, it is converted to NADH by electron transfer. Research indicates a decline in NAD^+^ levels during the natural aging process in both mice and humans, with changes in NAD^+^ metabolism intricately linked to aging [106]. Moreover, NAD^+^ is an essential cofactor for the deacetylase SIRT3, which regulates the acetylation status of mitochondrial proteins. Age‐related NAD^+^ depletion leads to reduced SIRT3 activity, impaired mitochondrial superoxide dismutase 2 activity, diminished antioxidant capacity, and weakened mitochondrial ETC function, forming a vicious cycle [107]. This suggests that supplementing with NAD^+^ precursors may provide new therapeutic opportunities for neurodegenerative diseases.

#### 2.2.5 Mitochondrial‐Driven Inflammation & Inflammaging

“Inflammaging” refers to the chronic, low‐grade inflammation that characterizes aging [108]. Recent research indicates that mitochondria is pivotal in driving this process. Dysfunctional mitochondria is not only the root cause of cellular energy metabolism disorders, but also an important source of DAMPs. By releasing a series of damage‐related molecular models, they initiate and continuously activate innate immune responses, thereby facilitating the onset and progression of inflammation, aging, and associated diseases [109, 110, 111].

One of the core mechanisms of mitochondrial inflammation is cytoplasmic leakage of mtDNA and its mediated activation of the cGAS‐STING pathway. During the aging process, due to the decline of mitochondrial autophagy function, increased mitochondrial membrane permeability, or disrupted mitochondrial cristae structure, mtDNA is more likely to leak from the mitochondrial matrix to the cytoplasm [112, 113, 114, 115]. The mtDNA in the cytoplasm is recognized by the pattern recognition receptor cGAS, which catalyzes the synthesis of the second messenger cGAMP. This molecule subsequently activates the STING protein and its downstream signaling pathways, including IRF3 and NF‐κB, leading to the production of Type I interferon and various proinflammatory cytokines [116]. Research has confirmed that abnormal activation of the cGAS–STING pathway is common in aging cells and tissues of elderly individuals. Enhancing mitochondrial autophagy through drugs or genetic means can effectively clear damaged mitochondria, reduce mtDNA leakage, inhibit this pathway, alleviate age‐related inflammation, and improve tissue function [112].

Mitochondria, in addition to mtDNA, can activate the NLRP3 inflammasome via various DAMPs and ROS. Under aging‐related metabolic stress, mitochondrial ROS production increases and promotes the assembly and activation of NLRP3 inflammasome, leading to caspase‐1 cleavage and the maturation and release of proinflammatory factors like IL‐1β and IL‐18 [117]. A positive feedback loop is formed between mitochondrial dysfunction and NLRP3 inflammasome activation, constantly amplifying inflammatory signals, especially in immune cells such as macrophages. This process significantly enhances the systemic inflammatory load and is closely related to aging‐related diseases such as neurodegenerative diseases, sarcopenia, and osteoarthritis [118, 119, 120].

Cellular senescence is closely associated with inflammation derived from mitochondria. In senescent cells, “permeabilization of a few outer mitochondrial membranes” frequently occurs, leading to the release of a small quantity of mtDNA. Although it is not enough to immediately trigger apoptosis, it is enough to drive the generation of senescence‐associated secretory phenotype through the cGAS–STING pathway, and then amplify the local and even systemic inflammatory response through paracrine action, promoting tissue aging and functional degradation [114].

**FIGURE 1:**
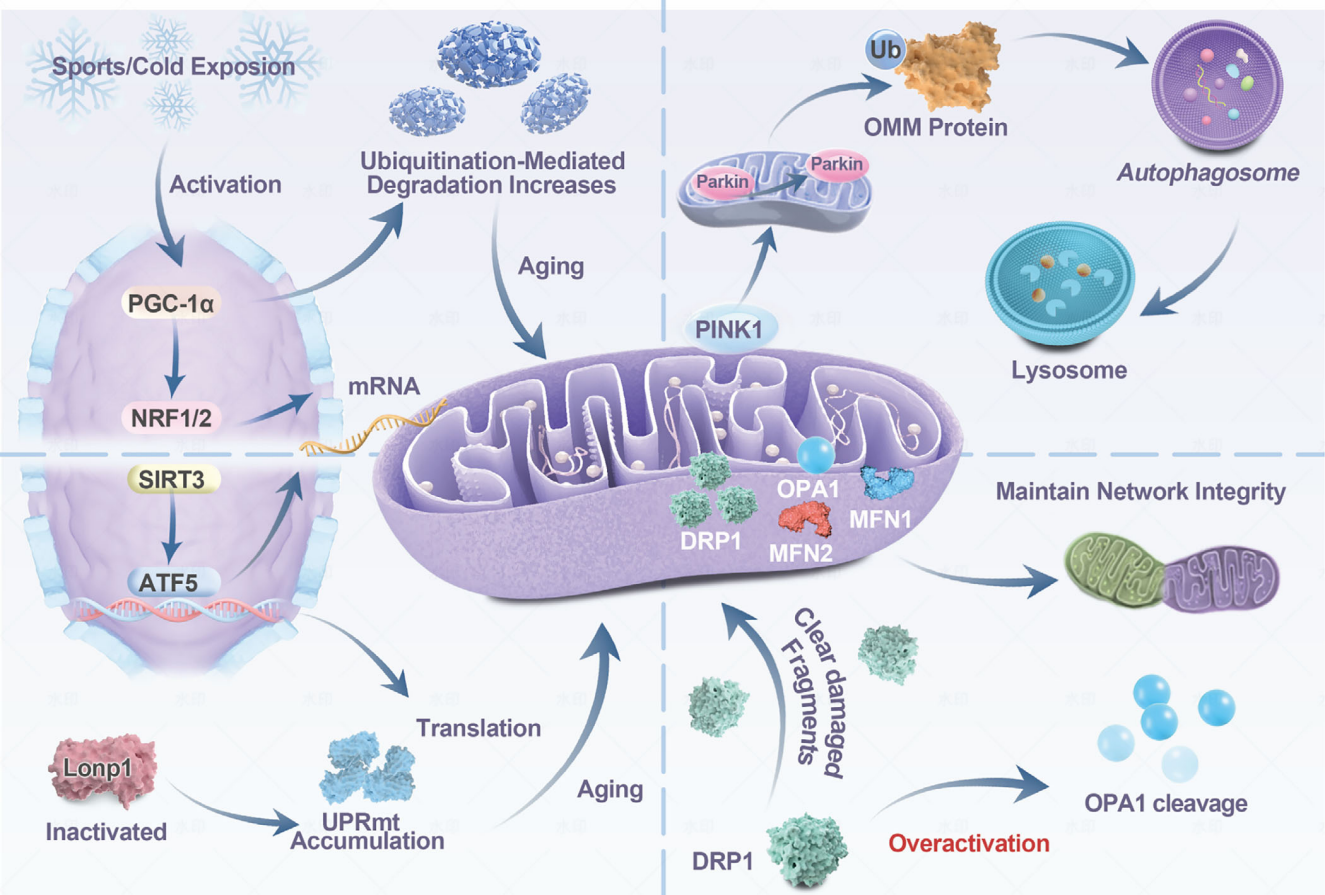
Overview of mitochondrial quality control mechanism. Mitochondrial quality control mechanism regulates mitochondrial function to maintain normal cell function. Mitochondrial quality control mainly includes four parts: mitochondrial autophagy, mitochondrial dynamics, mitochondrial unfolded protein reaction, and mitochondrial biogenesis. Mitochondrial autophagy (upper right) is mainly due to the activation of PINK1/Parkin pathway, which starts autophagy to wrap damaged mitochondria, and then is degraded by lysosomes. Mitochondrial dynamics (lower right) includes fusion and division, which are mediated by fusion protein MFN1/2, OPA1 and split protein DRP1, respectively. UPRmt (lower left) is a protective signaling pathway initiated by cells in response to mitochondrial protein folding abnormalities or dysfunction. When it is activated, the marker Lonp1 is upregulated. Mitochondrial biogenesis (upper left) refers to the process of the generation of new mitochondria. Exercise can activate PGC‐1α, thereby promoting the synthesis of mitochondria. ATF5: activating transcription factor 5 gene; NRF2: nuclear factor erythroid 2‐related factor 2.

As an important integration hub of cellular stress and immune response, mitochondria function disorder caused by mtDNA leakage, ROS overproduction and DAMPs release is a key molecular bridge connecting cellular senescence, systemic inflammation and aging‐related diseases. Targeting mitochondrial quality control, inhibiting the release of specific DAMPs, or blocking their downstream inflammatory pathways have become potential therapeutic strategies to alleviate inflammatory aging and delay the progression of aging‐related diseases (Figure 1).

## 3 Mitochondrial Dysregulation in Aging‐Related Diseases

The complex interplay between mitochondrial dysfunction and the pathogenesis of age‐related diseases constitutes a fundamental aspect of contemporary geroscience. The systemic decline in mitochondrial quality control, bioenergetic capacity, and redox homeostasis not only speeds up aging but also acts as a significant contributor to multisystem morbidity. This section delineates the organ‐specific manifestations of mitochondrial dysregulation across key physiological systems, highlighting novel mechanistic insights and emerging therapeutic paradigms (Table 1)

**TABLE 1:** Summary of mitochondrial dysregulation in major aging‐related diseases.

Disease category	Representative diseases	Key mitochondrial defects	Key molecular mechanisms/pathways	Major clinical/pathological impacts	References
Musculoskeletal disorders	Sarcopenia	Decreased ATP synthesis Excess production of ROS Accumulation of mtDNA mutations Impaired mitochondrial autophagy	Mitochondrial dynamics imbalance PINK1/Parkin pathway inhibition Calcium homeostasis dysregulation	Decreased muscle strength Muscle atrophy Reduced physical ability	[121]
	Osteoporosis	Increased osteoblast apoptosis Collapse of mitochondrial membrane potential Enhanced mitochondrial fission	Decreased OPA1 expression Defective mitochondrial autophagy mediated by PINK1/Parkin AOPPs‐induced NOX–ROS–mPTP apoptosis pathway	Reduced bone mass Increased risk of fractures Destruction of bone microstructure	[122, 123]
Neurological disorders	AD	Impaired oxidative phosphorylation (especially Complex IV) Decreased mtDNA copy number Fragmented mitochondrial morphology	Aβ inhibits ETC complexes Tau protein interferes with mitochondrial transport and dynamics UPRmt and mitophagy are transcriptionally upregulated	Cognitive decline Neuron loss Aβ plaques and Tau tangles	[124, 125, 126]
Neurological disorders	PD	Complex I activity‐specific defect mtDNA deletions/mutations impaired mitophagy	PINK1/Parkin mutations lead to mitochondrial autophagy dysfunction α‐Synuclein aggregation interacts with the mitochondrial import machinery	Loss of dopaminergic neurons Motor dysfunction Lewy body formation	[127, 128]
	HD/ALS	Decline in respiratory function Inhibition of mitochondrial autophagy (HD) Mitochondrial axonal transport disorder (ALS)	mHTT interferes with autophagy receptors (HD) SOD1 mutation causes oxidative stress (ALS) Downregulation of the Nrf2/ARE pathway	Striatal neuron degeneration (HD) Motor neuron death (ALS)	[129, 130]
Cardiovascular disorders	Heart failure	Reduced mitochondrial biogenesis Insufficient ATP production Abnormal mtDNA 6 mA methylation	NAD^+^ depletion → Z‐DNA accumulation → ZBP1‐mediated PANoptosis METTL4‐mediated abnormal mtDNA methylation	Decreased cardiac pumping function Increased risk of atrial fibrillation Metabolic remodeling	[131, 132]
	Atherosclerosis	Increased mtROS production Cytoplasmic mitophagy	cGAS‐STING pathway activation NLRP3 inflammasome activation Telomere shortening → p53/p21 senescence pathway	Chronic vascular inflammation Plaque formation and instability Endothelial dysfunction	[133, 134, 135]
Metabolic diseases	Type 2 diabetes	β‐Cell oxidative phosphorylation capacity decreases Loss of mitochondrial plasticity Impaired mitochondrial autophagy	Glucotoxicity and lipotoxicity Blocked insulin signaling pathway Mitochondrial ROS promotes insulin resistance	Insulin secretion defects Hyperglycemia Peripheral tissue insulin resistance	[136, 137]
	NAFLD/NASH	Decreased mitochondrial β‐oxidation efficiency in hepatocytes Accumulation of toxic lipid intermediates Excessive production of ROS	Abnormal splicing of DRAK2–SRSF6 PNPLA3–I148M polymorphism leads to mitochondrial dysfunction Decline in PRDX1 antioxidant function	Fatty liver degeneration Inflammation and fibrosis Progression to cirrhosis	[138, 139]

Abbreviations: AD, Alzheimer's disease; ALS, amyotrophic lateral sclerosis; HD, Huntington's disease; PD, Parkinson's disease.

### 3.1 Musculoskeletal Disorders

Age‐related musculoskeletal degeneration is intrinsically linked to mitochondrial dysfunction, impacting both skeletal muscle and bone homeostasis. The integrity of the musculoskeletal system is paramount for functional independence, and its decline significantly contributes to frailty and reduced quality of life in the elderly.

#### 3.1.1 Sarcopenia and Mitochondrial Energetics

Sarcopenia, defined as the gradual decline in skeletal muscle mass and strength, is a key aspect of aging [140, 141]. The efficacy of skeletal muscle, a tissue with high metabolic demand, is heavily dependent on mitochondrial ATP production. Age‐associated mitochondrial dysfunction manifests as impaired oxidative phosphorylation, reduced ATP synthesis, and a significant elevation in ROS production [142].

The bioenergetic deficit is intensified by the accumulation of mtDNA mutations and a decline in mtDNA copy number, which compromise the expression of critical ETC components [143]. The ensuing energy crisis disrupts calcium handling, impairs excitation‐contraction coupling, and diminishes muscle contractility. Furthermore, dysfunctional mitochondria activate proteolytic pathways, including the ubiquitin–proteasome system and autophagy–lysosome machinery, leading to accelerated protein degradation and muscle atrophy [141]. Recent evidence underscores the role of mitochondrial‐derived peptides and exosomes from young stem cells in mitigating these effects by restoring mitochondrial dynamics and function, offering novel avenues for therapeutic intervention [144].

At the same time, Ca^2+^ is the core messenger of excitation contraction coupling (EC) in skeletal muscle, and the precise regulation of its homeostasis is essential to maintain muscle function. In aging muscle, the phenomenon of impaired EC coupling function has been demonstrated, which will lead to the reduction of Ca^2+^ ions supplied to contractile elements, thereby reducing muscle strength [145]. These findings are connected to earlier research on the impairment of Ca^2+^ release in muscle and dysfunction of ryanodine receptor in the sarcoplasmic reticulum (SR) with reduced Ca^2+^ release due to aging [146]. In addition, the reduction of mitochondrial SR coupling structure also means that the perception of Ca^2+^ content by mitochondria is reduced, which may lead to the reduction of metabolic efficiency and thus the decline of skeletal muscle performance [141, 145].

#### 3.1.2 Osteoporosis and Osteoblast Dysfunction

Osteoporosis, a metabolic bone disorder with heightened bone resorption over formation, is profoundly influenced by mitochondrial integrity within bone cells [147]. The aging of BMSCs hampers their ability to differentiate into bone‐forming cells, a shift exacerbated by mitochondrial compromise [148, 149].

In osteoblasts, aging promotes an imbalance in mitochondrial dynamics, favoring excessive fission mediated by Drp1 hyperactivation [150]. Consequently, this causes mitochondrial fragmentation, increased ROS, decreased membrane potential, and ultimately, impaired differentiation and augmented apoptosis [151]. The inner mitochondrial membrane fusion protein OPA1, crucial for cristae organization and metabolic efficiency, is downregulated with age, further contributing to dysfunction [152]. Concurrently, defects in mitophagy, particularly through the PINK1/Parkin pathway, result in the accumulation of defective mitochondria [153]. These organelles release proapoptotic molecules like cytochrome *c*, triggering osteoblast death and undermining bone formation [154]. Emerging research highlights the significance of UPRmt in maintaining osteoblast viability and identifies its disruption as a critical factor in age‐related bone loss. Novel strategies targeting mitochondrial proteostasis, such as enhancing NAD^+^ bioavailability to activate sirtuins, are showing promise in reversing these deficits.

### 3.2 Neurological Disorders

The brain is the organ with the highest energy demand in the human body. Although its weight only accounts for about 2% of the body weight, it consumes 20% of the oxygen and glucose in the whole body [155]. That means the central nervous system's high energy demand makes it exceptionally vulnerable to mitochondrial insufficiency. Neuronal survival, synaptic plasticity, and cognitive function are critically dependent on optimal mitochondrial performance, whose age‐related decline is a cornerstone of neurodegenerative pathologies [156, 157, 158].

Neurodegenerative diseases linked to with aging include AD, PD, Huntington's disease (HD) and amyotrophic lateral sclerosis (ALS) [159]. The mechanisms underlying the progression of these diseases typically involve eight key features: pathological protein aggregation, dysfunction of synaptic and neuronal networks, abnormal protein homeostasis, abnormal cytoskeleton, alterations in energy homeostasis, defects in DNA and RNA, inflammation, and neuronal cell death [160].

#### 3.2.1 Alzheimer's Disease

AD stands out as the most prevalent neurodegenerative disease. Research indicates that the key neuropathological features of AD involve extracellular amyloid β‐protein (Aβ) plaques and intracellular hyperphosphorylated tau protein neurofibrillary tangles. Beyond these canonical markers, mitochondrial dysfunction is increasingly acknowledged as a primary factor in the development of AD. Postmortem analyses and neuroimaging studies in AD patients reveal consistent deficits in glucose metabolism and oxygen consumption, indicative of impaired oxidative phosphorylation. Morphologically, AD neurons exhibit abnormally small, fragmented mitochondria with disrupted cristae and increased osmophilic inclusions [12, 161].

A specific deficiency in cytochrome *c* oxidase activity has been frequently documented [162, 163]. The mitochondrial cascade hypothesis suggests that initial mitochondrial dysfunctions, potentially driven by mtDNA mutations or oxidative insult, promote amyloid precursor protein processing into Aβ, which in turn further damages mitochondria, creating a vicious cycle. Aβ oligomers can inhibit ETC complexes, depolarize mitochondrial membranes, and induce ROS production.

Furthermore, impaired mitochondrial dynamics disrupt the equitable distribution of organelles along axons, compromising synaptic function. Recent research has highlighted the essential role of mitochondrial quality control in AD. Upregulation of UPRmt and mitophagy‐related transcripts in early AD stages may represent a compensatory mechanism that ultimately becomes overwhelmed, contributing to neuronal failure [164, 165, 166].

In conclusion, numerous studies have highlighted the significant role of mitochondria in the pathogenesis of AD. The question of whether mitochondrial dysfunction is merely a consequence of amyloid deposition or if it directly contributes to the disease's initial stages remains contentious, necessitating further research and experimentation for clarification.

#### 3.2.2 Parkinson's Disease

PD is characterized by the degeneration of dopaminergic neurons in the substantia nigra and the presence of Lewy bodies that contain aggregated α‐synuclein [167]. Clinically, PD manifests through various combinations of bradykinesia, rigidity, resting tremor, and postural instability, along with additional motor and nonmotor symptoms [168].

A hallmark of PD is a specific deficiency in Complex I activity, which can be recapitulated by toxins like MPTP and rotenone [169, 170]. Genetic studies firmly establish the role of mitochondria, as mutations in PINK1 and Parkin, which are crucial for mitophagy, cause autosomal recessive early‐onset PD. These mutations hinder the selective removal of damaged mitochondria, leading to their accumulation and subsequent neuronal toxicity [171].

Furthermore, deletions of mtDNA and reductions in copy number are prevalent among patients with PD [172]. A detrimental feedback loop exists between mitochondrial dysfunction and α‐synuclein pathology: dysfunctional mitochondria promote α‐synuclein aggregation, and pathologic α‐synuclein can bind to and impair the mitochondrial import machinery, disrupt dynamics, and enhance ROS production. Current research is exploring mitochondrial transplantation and compounds that boost PINK1/Parkin signaling as potential disease‐modifying therapies [173].

#### 3.2.3 HD and ALS

HD arises from the expansion of CAG trinucleotide repeats that encode polyglutamine (poly Q) clusters in the amino terminal segment of huntingtin (HTT) [174]. Although mutant HTT (mHTT) is ubiquitously expressed, this disease is characterized by preferential atrophy of the striatum due to the loss of GABAergic medium‐sized spiny neurons in the caudate nucleus and putamen, and other brain regions such as the cerebral cortex are also affected [175].

In HD, mitochondrial dysfunction is one of the core pathological mechanisms driving the occurrence and development of disease. Its core feature is that the poly Q amplification of mHTT leads to significant inhibition of the mitophagy pathway. At the same time, mHTT also interacts with autophagy receptors, hindering the formation and encapsulation of autophagosome membranes. Due to the blocked mitophagy, a large number of functionally impaired mitochondria accumulated in mHTT cells. This leads to decreased respiratory function, reduced ATP production, and elevated levels of ROS, leading to energy crisis and oxidative stress, which ultimately selectively exacerbate the vulnerability and degeneration of medium spiny neurons in the striatum [176].

ALS is a prevalent and intricate neurodegenerative disorder characterized by the progressive loss of upper and lower motor neurons. The gradual deterioration of limbs leads to muscle wasting, paralysis, and eventual death within 3–5 years of onset [177]. In ALS, mitochondrial dysfunction is involved in the specific degeneration of motor neurons, especially in the transport disorder and morphological abnormalities of mitochondria in axons.

Mitochondrial dysfunction contributes to the selective death of motor neurons in ALS. Mutations in SOD1 disrupt redox homeostasis, while impaired mitochondrial axonal transport and abnormal morphology are commonly observed [177]. A noted deficiency in the Nrf2/ARE antioxidant pathway further exacerbates oxidative damage, making enhancing mitochondrial antioxidant defenses a promising therapeutic angle for these diseases [178].

### 3.3 Cardiovascular System Aging

Cardiovascular diseases (CVDs) persist as a prominent cause of death, with aging being a key risk factor. This heart's relentless energy demands render cardiomyocytes particularly dependent on robust mitochondrial function, making this organ highly susceptible to age‐related mitochondrial decay.

As aging progresses, the heart will undergo various metabolic and structural changes, which will eventually manifest as HF and atherosclerosis (AS). Aging of different cell types in the heart together constitutes cardiac aging, involving cardiomyocytes, fibroblasts, endothelial cells, immune cells, and so on [179, 180]. Researchers have identified macroautophagy dysfunction, loss of protein homeostasis, genomic instability, epigenetic changes, mitochondrial dysfunction, cellular senescence, dysregulation of neurohormonal signaling, and inflammation are recognized as prevalent molecular indicators of cardiovascular aging [181]. Among these factors, mitochondrial dysfunction stands out as the primary contributor to cardiovascular aging.

#### 3.3.1 HF and Metabolic Remodeling

HF, particularly HF with preserved ejection fraction (HFpEF), being notably linked to substantial mitochondrial abnormalities. These include impaired mitochondrial biogenesis, decreased oxidative capacity, and inefficient ATP production [182]. A groundbreaking recent discovery links mitochondrial dysfunction in HFpEF‐related atrial fibrillation to the accumulation of Z‐DNA within mitochondria [183]. This occurs due to NAD^+^ depletion and the subsequent downregulation of mitochondrial topoisomerases [184]. Z‐DNA is recognized by Z‐DNA binding protein 1 (ZBP1), which initiates a unique form of programmed cell death termed PANoptosis, which promotes atrial electrical remodeling and fibrillation [185]. Notably, supplementation with NAD^+^ precursors like β‐NMN can restore mitochondrial homeostasis and suppress this pathway, revealing a potent therapeutic target [186, 187]. Another emerging mechanism involves the dysregulation of mtDNA methylation, specifically N6‐methyladenine, mediated by enzymes like METTL4, which contributes to mitochondrial dysfunction and HF pathogenesis, suggesting another avenue for intervention [132].

#### 3.3.2 AS and Vascular Inflammation

Previous studies have suggested that as is a degenerative disease caused by lipid accumulation and mechanical damage to the arterial wall [188]. With the development of molecular biology and systems medicine, AS is no longer viewed merely as a lipid storage disease but as a chronic inflammatory process driven by aging mechanisms, with mitochondrial dysfunction at its core [189]. In aged vasculature, mitochondria exhibit swollen morphology, reduced cristae, increased ROS production (mtROS), and impaired oxidative phosphorylation. These common features indicate that aging and as are not independent phenomena, but are closely linked through overlapping molecular pathways [190].

During vascular aging, cellular mitochondria undergo significant structural and functional decline, manifested by morphological changes such as mitochondrial swelling and decreased cristae structure, accompanied by decreased oxidative phosphorylation capacity, insufficient ATP synthesis, and increased mtROS production [191, 192, 193]. The resulting mtROS exacerbates mtDNA damage, and this damaged mtDNA can be released into the cytoplasm. Acting as a DAMP, it triggers innate immune sensors like TLR9 and the cGAS–STING pathway, culminating in the assembly of the NLRP3 inflammasome and the production of potent proinflammatory cytokines like IL‐1β and IL‐18 [194, 195]. Consequently, a detrimental cycle of chronic inflammation ensues, promoting endothelial dysfunction, recruitment of leukocytes, and formation of plaques.

Furthermore, age‐related NAD^+^ depletion reduces the activity of SIRT1 and SIRT3, diminishing their anti‐inflammatory and antioxidant effects [193], while impaired PINK1/Parkin‐mediated mitophagy allows damaged, ROS‐producing mitochondria to persist. Telomere shortening in vascular cells activates p53/p21 signaling, inducing a senescent, proinflammatory, and prothrombotic phenotype that further accelerates vascular aging and AS [196].

### 3.4 Metabolic Diseases

#### 3.4.1 Insulin Resistance and Type 2 Diabetes

Mitochondria serve as essential regulators of cellular metabolism, and their dysfunction is a common thread linking age‐related metabolic disorders such as obesity, Type 2 diabetes (T2D), and nonalcoholic fatty liver disease (NAFLD) [197, 198, 199].

Studies have shown that brown adipose tissue mitochondria are rich in uncoupling protein 1 (UCP1), enhancing the proton conductance of the inner mitochondrial membrane, dissipates the proton gradient, uncouples oxidative phosphorylation from ATP production, and thus converts the energy generated by substrate oxidation into heat [200, 201]. The thermogenic mechanism helps the body resist hypothermia, obesity, and associated metabolic disorders. White adipose tissue (WAT) is the main energy reservoir in mammals; the lack or impairment of WAT results in the abnormal accumulation of fats in alternate tissues, like the liver [202]. In physiological conditions, wat adipocytes can efficiently deliver mitochondria to macrophages through intercellular mitochondrial transfer, but this process is notably impaired under obesity. Heparan sulfate serves as a crucial regulator of mitochondrial uptake by macrophages. Mice with macrophage‐specific deletion of heparan sulfate demonstrate diminished energy metabolism, heightened vulnerability to obesity, and insulin resistance [203; 204]. These findings highlight the significance of intercellular mitochondrial transfer in preserving metabolic balance, with its impairment potentially contributing to obesity.

Pancreatic β‐cell dysfunction is central to T2D development [205]. Mitochondrial oxidative phosphorylation is critical for coupling glucose metabolism with insulin secretion [206]. In T2D, β‐cells often exhibit reduced mitochondrial mass, oxidative capacity, and metabolic flexibility. This impairment can be both acquired and genetic. Excessive mitochondrial ROS contributes to β‐cell dysfunction and insulin resistance in peripheral tissues [207]. Recent studies highlight that proteins like DRAK2 can inhibit mitophagy through the phosphorylation and degradation of ULK1, disrupting mitochondrial turnover and compromising β‐cell health during diabetic stress [208]. Therapeutic strategies aimed at improving mitochondrial quality and function in β‐cells and skeletal muscle are actively being pursued.

#### 3.4.2 NAFLD/Nonalcoholic Steatohepatitis and Hepatic Mitochondria

**FIGURE 2:**
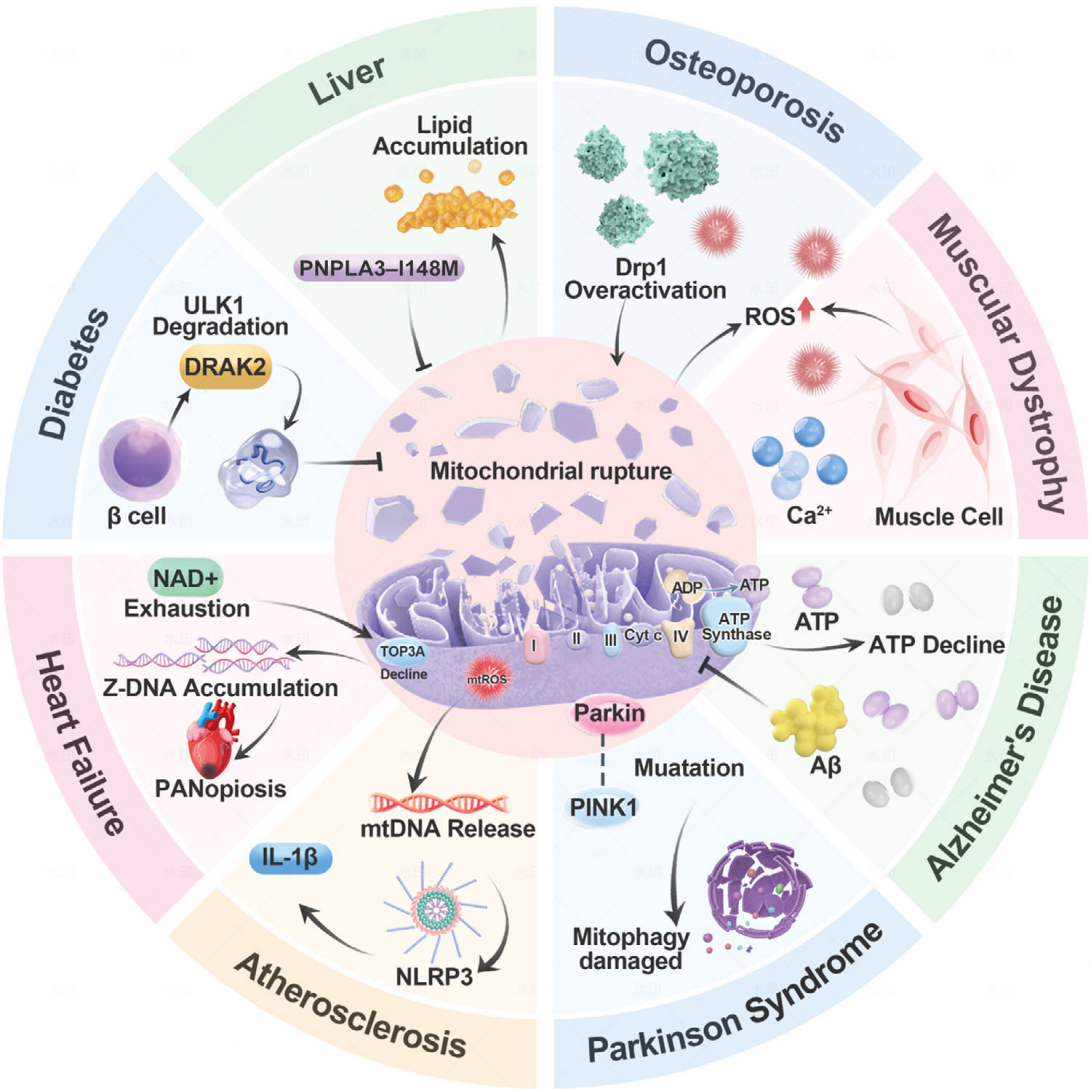
Mitochondrial disorders in aging‐related diseases. With age, the decline of mitochondrial quality control system leads to mtDNA damage, ROS imbalance, calcium homeostasis disorder, metabolic abnormalities, and mitochondrial driven chronic inflammation and other dysfunction. These dysfunction further aggravate cell aging and specifically drive pathological changes in different tissue systems, including musculoskeletal diseases such as osteopenia and osteoporosis, neurodegenerative diseases such as Alzheimer's disease and Parkinson's disease, cardiovascular diseases such as heart failure and atherosclerosis, and metabolic diseases such as Type 2 diabetes and nonalcoholic fatty liver disease. ULK1: unc‐51 like kinase 1 gene; DRAK2: death‐associated protein‐related apoptotic kinase 2; PNPLA3–I148M: patatin‐like phospholipase domain‐containing 3, I148M is a mutation of this gene.

Obesity and other factors lead to abnormal accumulation of lipids in nonadipose tissues, such as the liver, which can trigger NAFLD [209]. The NAFLD spectrum includes simple steatosis, steatohepatitis, liver fibrosis, and ultimately cirrhosis [210]. Research shows that, the progression from simple hepatic steatosis to nonalcoholic steatohepatitis (NASH) involves significant mitochondrial dysfunction [211]. While initial steatosis may involve efficient fatty acid β‐oxidation, advanced NASH is marked by ineffective oxidation, resulting in the accumulation of harmful lipid intermediates and increased ROS production [212]. Recent research implicates dysregulated RNA splicing of mitochondrial genes in NASH progression. For instance, DRAK2 can phosphorylate the splicing factor SRSF6, leading to aberrant splicing of key mitochondrial genes, which impairs mitochondrial function and drives hepatic lipid accumulation [213]. Furthermore, the antioxidant protein peroxiredoxin 1 (PRDX1) plays a protective role by scavenging H_2_O_2_ and mitigating mitochondrial oxidative stress, thereby slowing NASH progression [214]. Additionally, genetic variants like PNPLA3–I148M disrupt mitochondrial function in hepatocytes, impairing fat utilization and ketogenesis, which promotes NAFLD development. These discoveries introduce novel avenues for precision therapies targeting the rectification of splicing abnormalities or the improvement of mitochondrial oxidative equilibrium (Figure 2).

## 4 Mitochondria Targeted Therapeutic Strategies

Mitochondrial dysfunction has been recognized as a critical mechanism driving the occurrence and development of aging and related diseases. Its functional decline promotes the aging process and tissue function degradation of the body through multiple pathways such as aggravating oxidative stress, destroying energy metabolism, disturbing dynamic balance, and triggering chronic inflammation. Therefore, research on targeted therapeutic strategies that can directly intervene and correct these defects is not only crucial, but also shows great potential for clinical transformation (Table 2).

**TABLE 2:** Mitochondria‐targeted therapeutic agents: mechanisms and evidence.

Strategy	Compound	Mechanism	Clinical evidence	References
Mitochondria‐targeted antioxidants	Mitoquinol mesylate (MitoQ)	TPP^+^‐linked CoQ derivative; accumulates in matrix 100–500×; reduces ROS, improves vascular function	25+ human trials; improves endothelial function, energy metabolism	[20, 215, 216, 217]
	SkQ1	TPP+‐linked plastoquinone; protects mtDNA; delays aging in accelerated aging models	Approved for dry eye disease; antiaging trials ongoing	[218, 219, 220]
	SS‐31(Elamipretide)	Cell‐penetrating peptide; binds to cardiolipin, stabilizes ETC supercomplexes	Phase II/III trials for mitochondrial myopathies	[221, 222, 223, 224, 225]
	MitoTEMPO	TPP+‐linked SOD mimetic; reduces mitochondrial superoxide, improves redox balance	Preclinical models of metabolic and renal disease	[226, 227]
	N‐acetylcysteine	Supplement glutathione precursors to enhance the overall antioxidant capacity of cells	Improving the senescence phenotype of aged fibroblasts and osteoarthritic chondrocytes	[228]
	Glutathione	Directly supplement key antioxidants	Can protect the chondrogenic phenotype in chondrocytes without impairing cell proliferation or matrix synthesis	[229]
Indirect antioxidants	Resveratrol	Activate SIRT1, deacetylate and activate PGC‐1α, and upregulate antioxidant enzymes such as SOD and catalase	In mice on a high‐fat diet, it can improve health and extend lifespan	[230, 231, 232]
	NMN	Increase NAD^+^ levels, activate SIRT1, SIRT3, and others, enhance deacetylase activity and antioxidant defense	Chronic supplementation with nicotinamide riboside is safe and can efficiently promote NAD^+^ levels.	[233, 234, 235]
Mitophagy inducers	Trehalose	mTOR‐independent autophagy/mitophagy activator	In a mouse model with age‐related neurological symptoms caused by Atg7 knockdown, it can delay the aging process.	[236, 237]
	Urolithin A	Induce mitophagy to remove dysfunctional mitochondria	Long‐term treatment in AD model mice can restore lysosomal function and reverse cognitive, olfactory, and synaptic plasticity impairments.	[238, 239]
	Lithium	Mitophagy inducer	Extend the healthspan without altering the mortality curve	[240, 241]
Nrf2 pathway activators	Sulforaphane	Inactivate Keap1, stabilize Nrf2, and translocate it to the nucleus to activate antioxidant response elements	Shown broad cytoprotective effects in various disease models	[242]

### 4.1 Generalized Intervention Strategies

#### 4.1.1 Antioxidants

Mitochondria, being the main origin of intracellular reactive ROS, are pivotal in the aging process and the development of age‐related illnesses [243]. The buildup of mitochondrial oxidative harm drives cellular senescence, tissue dysfunction, and systemic aging phenotypes [244, 245]. While traditional antioxidants have shown limited efficacy due to poor bioavailability and lack of mitochondrial specificity, recent progress in therapies targeting antioxidants to mitochondria presents encouraging approaches to alleviate oxidative stress at its root [246, 247].

MitoQ, a widely researched mitochondrial antioxidant, employs a triphenylphosphonium (TPP^+^) cation coupled to a modified CoQ derivative [248]. This structure enables it to cross lipid membranes and allows it to accumulate in the mitochondrial matrix at concentrations several hundred times greater than in the surrounding environment.

In neurodegenerative models, MitoQ significantly reduced mitochondrial ROS production, improved mitochondrial ultrastructure, and decreased oxidative stress‐induced cell death [249]. Human clinical evidence continues to emerge; studies in individuals aged 60–79 years showed that 20 mg daily MitoQ supplementation for 6 weeks improved arterial dilation by 42%—equivalent to reducing vascular age by 15–20 years [20]. Notably, MitoQ achieved this at approximately 1/50th the dosage required for conventional CoQ10 to produce similar effects.

SkQ1, another targeted antioxidant utilizing the TPP^+^ delivery system, has demonstrated remarkable antiaging properties in animal models [250]. Research using mutation‐prone mice with accelerated aging showed that SkQ1 supplementation extended average lifespan by approximately 15%, significantly delayed the onset of age‐related phenotypes, and preserved mitochondrial structure in cardiac and hepatic tissues [251].

Recent advances in antioxidant therapeutics emphasize multimodal and targeted strategies that move beyond single‐agent interventions. Conventional antioxidants such as N‐acetylcysteine and glutathione exhibit mitochondrial relevance [252]. N‐acetylcysteine, as a glutathione precursor, ameliorates senescence‐associated phenotypes in aged fibroblasts and chondrocytes but may compromise extracellular matrix synthesis during cartilage repair [253, 254]. In contrast, glutathione supplementation preserves chondrogenic identity without adverse effects on proliferation or matrix production [255]. A more refined approach is exemplified by SS‐31, a tetrapeptide targeted to mitochondria that builds cardiolipin in the inner mitochondrial membrane, stabilizes ETC supercomplexes, enhances electron transfer efficiency, and selectively neutralizes hydroxyl and peroxynitrite radicals [24]. This mechanism has conferred neuroprotection in preclinical models of AD and PD, underscoring the value of subcellular targeting in redox therapeutics [256].

Growing evidence supports the superiority of combined antioxidant regimens over monotherapy. Studies in murine models demonstrate that mixed antioxidant formulations significantly enhance spatial cognition, short‐term memory, and muscular endurance [257]. These synergistic effects likely arise from the complementary actions of individual components on distinct nodes of the oxidative stress network, ranging from radical scavenging to metal chelation and enzyme modulation, thereby offering more robust and holistic defense against age‐related functional decline. Such combinatorial strategies reflect a paradigm shift toward systems‐level redox regulation rather than isolated ROS quenching.

An alternative and increasingly compelling approach involves indirect antioxidant strategies that bolster endogenous defense mechanisms. Activation of sirtuins, particularly the NAD‐dependent deacetylase SIRT1, enhances mitochondrial resilience by deacetylating and activating PGC‐1α, which in turn upregulates key antioxidant enzymes like catalase, glutathione peroxidase, and manganese superoxide dismutase [258, 259]. In neuronal contexts, SIRT1 activation mitigates mitochondrial ROS and prevents oxidative stress‐induced apoptosis. Natural activators like resveratrol further validate this pathway's therapeutic relevance [260]. Additional sirtuins, including SIRT2 and SIRT3, also contribute to mitochondrial redox homeostasis [261]. Parallel efforts focus on mitophagy induction using agents such as trehalose and lithium to selectively eliminate dysfunctional, ROS‐producing mitochondria. These compounds reduce superoxide burden and alleviate age‐associated conditions like AS. Notably, lithium extends healthspan in Caenorhabditis elegans without affecting lifespan, while trehalose rescues neurodegeneration in autophagy‐deficient mice, highlighting the pivotal importance of mitochondrial quality control in aging and disease [262, 263].

Despite promising preclinical findings, the clinical application of mitochondrial antioxidant treatments encounters enduring obstacles. One major obstacle is achieving sufficient bioavailability and precise tissue‐specific mitochondrial targeting, as many compounds struggle to reach therapeutically effective concentrations within mitochondria of affected organs [264]. Although TPP‐conjugated molecules demonstrate efficient mitochondrial accumulation, their uneven biodistribution across tissues can limit efficacy in certain contexts. Additionally, dosing and timing are critical considerations: reactive ROS serve dual roles as both cytotoxic agents and essential signaling molecules, meaning that indiscriminate suppression may impair vital redox signaling pathways [265]. An ideal therapeutic strategy would selectively mitigate pathological oxidative stress without disrupting physiological redox homeostasis. Furthermore, substantial interindividual variability—driven by genetic background, nutritional status, environmental exposures, and disease stage—underscores the need for personalized approaches. Identifying reliable biomarkers to predict treatment response will be essential for tailoring antioxidant regimens to individual patients [266].

The future of mitochondrial antioxidant therapy is likely to center on integrated, multimodal strategies that concurrently address multiple facets of mitochondrial dysfunction. Combining direct antioxidants with agents that enhance mitophagy, stimulate mitochondrial biogenesis, and modulate inflammation may yield synergistic benefits, better matching the complex, multifactorial nature of age‐related mitochondrial decline. Beyond pharmacological combinations, emerging technologies are poised to redefine the field. These include mitochondrial transplantation, gene‐editing techniques aimed at boosting endogenous antioxidant systems, and advanced nanocarriers engineered for precise mitochondrial delivery [267]. As these innovations mature, they hold the potential to overcome current pharmacokinetic and specificity limitations, offering more robust and durable interventions to preserve mitochondrial integrity and function across the lifespan.

#### 4.1.2 Metabolic Regulators

Compared with targeted or active delivery of antioxidants to alleviate ROS‐mediated mitochondrial damage, a more fundamental strategy is to positively regulate mitochondrial function itself. The core goal of this strategy is to inhibit the generation of ROS from the source on the premise of maintaining sufficient ATP synthesis.

Mitochondrial ROS is mainly produced by Complex I in forward and reverse electron transfer and Complex III, and its yield is positively related to MMP, and is extremely sensitive to changes in membrane potential. There is a nonlinear relationship between the two [268]. Mitochondrial uncoupling refers to the process of proton leakage through mitochondrial inner membrane, thus bypassing ATP synthase [269]. This process makes the proton power generated by the ETC being insufficient to fully drive ATP synthesis. Therefore, mild mitochondrial uncoupling induced by endogenous pathways such as UCPs or chemical uncoupling agents can effectively dissipate membrane potential, thereby significantly inhibiting ROS generation [270]. According to the hypothesis of “uncoupling for survival,” moderate uncoupling under the premise of maintaining sufficient ATP production can reduce oxidative damage and possibly extend life span.

Congenital mitochondrial uncoupling arises through specific proteins integrated into the inner mitochondrial membrane. UCPs represent a crucial group of regulatory proteins that localize to inner mitochondrial membrane. Its classical mechanism of action facilitating the re‐entry of protons into the mitochondrial matrix autonomously from ATP synthase, thereby uncoupling the electron transport and ATP synthesis in the process of oxidative phosphorylation, enabling the dissipation of energy stored in the proton gradient as heat. This process is called uncoupled respiration [271, 272]. In addition to the uncoupling effect, UCPs can be activated by ROS and lipid peroxidation products, which can inhibit the overproduction of ROS from the source by moderately reducing the MMP, thus exerting the endogenous antioxidant defense function [273]. Recent research has shown that UCPs are widely involved in regulating whole‐body energy metabolism, involving multiple physiological and pathological processes such as insulin secretion, fatty acid metabolism, and neuroprotection, so they are regarded as key molecular hubs connecting energy metabolism, oxidative stress, and age‐related diseases [274, 275, 276, 277].

Genetic research has revealed overexpression of UCP2 in neurons can prolong the lifespan of Drosophila, accompanied by decreased ROS production. Meanwhile, mutant flies lacking the circadian transcriptional repressors Per and Tim were able to induce the UCP4C to extend lifespan [278]. Similarly, mice that specifically express UCP1 in skeletal muscle also exhibit increased lifespan and resistance to age‐related diseases such as AS [279, 280].

Unlike regulating the abundance or function of UCP in the naturally occurring mitochondrial uncoupling pathway, employing chemical uncouplers for mitochondrial uncoupling offers greater specificity by facilitating proton transport across the inner mitochondrial membrane and migration into the mitochondrial matrix from the intermembrane space, thereby diminishing the proton motive force [281, 282]. Consequently, inducing mild mitochondrial uncoupling with chemical uncouplers could offer a more robust evaluation of the “uncoupling survival” hypothesis.

The chemical uncoupler 2,4‐dinitrophenol (DNP) has been demonstrated to extend the lifespan of various model organisms, including yeast, Caenorhabditis elegans, Drosophila, and mice [283, 284, 285, 286]. However, the contradictions in the existing evidence about whether DNP effectively mediates life extension, and the conclusion is far from certain [284]. Although some studies have reported the positive effects of DNP on lifespan extension across different model organisms, the reproducibility and general applicability of these results are still a matter of debate [287]. At the same time, other studies have found that other chemical uncouplers, such as carbonyl cyanide‐3‐chlorophenylhydrazone and carbonyl cyanide‐p‐trifluoromethoxy phenyl hydrazine (FCCP), can also extend the life span of Caenorhabditis elegans. According to the existing evidence, it is still unclear whether mild uncoupling of traditional chemical uncoupling agents can extend the life span.

Chemical uncouplers with a broad therapeutic range are desirable, where a substantial rise in their concentration results in only a minimal increase in uncoupling. Butylated hydroxytoluene (BHT) has been shown to partially uncouple isolated rat mitochondria and rat thymocytes (2 × 10^−12^ M) in vitro even at very low levels, exhibiting more pronounced uncoupling effects than at the highest concentration tested (2 × 10^−6^ M) [288]. However, mice treated with BHT did not uncouple, and ROS production and overall oxidative protein damage were not significantly reduced, suggesting that the effects of chemical uncouplers on the isolation of mitochondria and cells in vitro may not always reflect similar effects in vivo [289]. Therefore, when determining whether chemical uncouplers can be used as antiaging drug interventions, the bioavailability, metabolism, therapeutic range, variability of therapeutic effective dose, distribution of organs and tissues in the body, toxic and side effects, and nonspecific effects of chemical uncouplers are important considerations [278, 289].

Novel mitochondria‐targeted uncouplers such as FCCP, niclosamide, and BAM15 have been developed to enhance the therapeutic safety of conventional uncouplers [290]. Studies have demonstrated that FCCP is very effective in dissipating mitochondrial proton gradients, thereby maximizing the stimulation of mitochondrial respiration. Maximal respiratory capacity was measured in the presence of the uncoupler niclosamide. Preincubation of FCCP had no effect on basal oxygen consumption rate, but inhibited the maximum respiration rate induced by niclosamide by about 30%. The irreversible inhibition of mitochondrial oxygen consumption by FCCP was confirmed [290]. Moreover, bam15 has been proved to be an effective and efficient mitochondrial uncoupler with potential for obesity treatment [291, 292].

#### 4.1.3 Regulators of Mitochondrial Dynamics

Mitochondria are not static organelles but maintain a dynamic equilibrium through division and fusion, resulting in a tightly controlled reticular system referred to as mitochondrial dynamics. As the central link of mitochondrial quality control, mitochondrial dynamics often appear significant imbalance during aging, generally showing enhanced division and weakened fusion [42]. This dysregulation results in fragmentation of the mitochondrial network, impaired function, and an exacerbation of oxidative stress levels, ultimately promoting the activation of apoptosis pathways, which has become an important pathological basis for various aging‐related disorders, like neurodegenerative diseases, CVDs, and metabolic diseases [293, 294, 295]. Consequently, targeted modulation of mitochondrial dynamics and remodeling of its network balance are regarded as a promising generalized intervention strategy.

Mitochondrial fission is mainly mediated by Drp1. In aging and related diseases, the activity of Drp1 or its recruitment to mitochondria is often abnormally upregulated. Therefore, the development of Drp1 inhibitors is a hot direction in regulating dynamics. Mitochondrial fission inhibitor‐1 (Mdivi‐1), a nonspecific inhibitor of Drp1‐dependent mitochondrial fission, has neuroprotective effects in many preclinical disease models, including AD and rodent models of ischemic or traumatic brain injury [296].

While Mdivi‐1 is commonly utilized as a drp1 inhibitor, its mechanism of action does not involve direct inhibition of the GTPase activity of Drp1; instead, it may impact mitochondrial fission indirectly [297, 298]. More importantly, a large amount of research evidence shows that mdivi‐1 has many drp1‐independent targets in mammalian cells [296, 299, 300]. In recent years, studies have found that Alpinetin, a natural flavonoid, can improve aging‐related cognitive dysfunction by inhibiting drp1‐mediated excessive mitochondrial fission, thereby blocking the downstream mitochondrial inflammatory pathway. At the same time, they also found that mdivi‐1 combined with kaempferin can produce synergistic effects [301]. It provides solid preclinical evidence for the advancement of therapeutic approaches aimed at modulating mitochondrial dynamics.

A strategy that can complement fission inhibition involves boosting mitochondrial fusion. The fusion process is facilitated by MFN1/2 of the outer mitochondrial membrane and the OPA1 protein in the inner mitochondrial membrane, and both of which frequently exhibit reduced expression or functionality with aging. Notably, significant advancements has been achieved in the development of agonists targeting fusion proteins. A study found that S89, a derivative of Spiraea extract natural product, has the efficacy of promoting mitochondrial fusion, and this reaction is only effective in cells expressing endogenous MFN1 [302]. The results suggest that mitochondrial‐related diseases can be effectively and safely treated without genome editing. The rapid restoration of mitochondrial membrane dynamics imbalance by S89 is contingent upon the presence of functional mfn1. Consequently, the repair of mitochondrial impairment, encompassing membrane potential, redox homeostasis, and ATP generation, can avert programmed cell demise, such as iron‐induced apoptosis and apoptosis

Molecule MASM7 was recently reported to regulate the fusion activity of MFN2. MASM7 promotes mitochondrial fusion by activating MFN2 and causing its conformational change, thereby increasing MMP, boosting oxidative phosphorylation and ATP production, which is expected to alleviate metabolic‐related diseases caused by aging [42, 303]. For OPA1, strategies focus on stabilizing its protein form, such as increasing the level of functionally active long‐form OPA1 by inhibiting the activity of proteases responsible for cleaving OPA1, thereby stabilizing mitochondrial cristae structure and improving oxidative phosphorylation function [304]. In sarcopenia and skin aging, enhanced fusion has been shown to be a key way to maintain tissue function and combat aging‐related degradation [305, 306].

Simultaneously, the mechanism underlying the antiaging or cytoprotective effects of numerous compounds is closely associated with the modulation of mitochondrial dynamics. For example, in addition to its antioxidant and activating deacetylase SIRT1, resveratrol can deacetylate and activate OPA1 through a SIRT1‐dependent pathway, thereby promoting mitochondrial fusion and improving metabolic homeostasis. Additionally, antioxidants targeting mitochondria reduce the damage of oxidative stress to key kinetic proteins such as Drp1 and OPA1 by directly scavenging excess ROS in mitochondria, and indirectly maintain the balance of dynamics. In glaucoma models, treatment of mitochondrial dynamics disorders in retinal ganglion cells has demonstrated effectiveness in protecting the optic nerve and delay disease progression [307].

In conclusion, taking mitochondrial dynamics as the target and accurately balancing the process of division and fusion through small molecules or biological agents represents a new paradigm of root cause treatment against aging and related diseases. With the in‐depth understanding of the regulatory mechanism of dynamic protein structure and function and the accumulation of more preclinical and clinical data, drug development in this field is expected to open up a new path aimed at delaying aging and addressing a range of age‐related diseases.

#### 4.1.4 Strategies for Mitochondria‐Derived Inflammation

Mitochondria serve as both the cellular energy generator and a crucial modulator of innate immune responses [308, 309]. During aging, mitochondrial dysfunction, characterized by oxidative mtDNA damage and heightened mitochondrial membrane permeability, triggers the liberation of mitochondrial DAMPs into the cytosol. This aberrantly activates the cGAS‐STING pathway and NLRP3 inflammasome, fostering persistent, subclinical inflammation known as “inflammaging.” Therefore, targeting these pathways has become an important therapeutic strategy for retarding aging and associated disorders.

The cGAS–STING pathway is a core mechanism for sensing cytosolic DNA. Studies have shown that abnormal activation of this pathway has been detected in various tissues of aging individuals and aging models [310, 311, 312]. Inhibiting this pathway can effectively improve aging‐related pathological phenotypes. Several studies have confirmed the therapeutic potential of specific inhibitors. In the aging‐related endothelial dysfunction model, the application of RU.521, a cGAS‐specific inhibitor, can significantly improve vascular function. Other studies have confirmed that the inhibitor has a positive effect on neuronal apoptosis and microglia activation [313]. In the 5 × FAD mouse model of AD, STING inhibitor H‐151 can effectively inhibit the activation of cGAS–STING pathway in microglia, reduce neuroinflammation, AD pathology and improve cognitive function [314]. However, because STING inhibitors require repeated high‐dose intraperitoneal injections, their efficacy is limited. Long‐term systemic use may increase susceptibility to cancer and infections [315]. Consequently, one potential therapeutic strategy for STING‐driven diseases involves local or targeted inhibition of cGAS–STING.

In addition, Ginkgetin, an active ingredient extracted from Ginkgo biloba leaves, a traditional Chinese medicine, was confirmed to directly bind to sting protein to inhibit its activation, thereby alleviating systemic inflammation and cellular senescence in an aging mouse model [316]. These findings confirm the pivotal role of the cGAS–STING pathway in aging and offer concrete support for its potential as a therapeutic target. In addition to directly targeting cGAS–STING, regulating its upstream elements is also a feasible strategy. For example, studies have found that PINK1 deficiency can trigger the cGAS–STING pathway and accelerate renal aging by causing mitochondrial metabolism dysregulation and mtDNA cytosolic release [317]. Similarly, the decline of YAP/TAZ function in stromal cells can damage nuclear envelope integrity, lead to genomic DNA leakage and activate cGAS–STING, driving tissue senescence [318]. This process can be reversed by maintaining YAP activity or using STING inhibitors. This suggests that preventing the release of DAMPs by enhancing mitochondrial quality control and maintaining cytoskeletal integrity is an effective way to indirectly inhibit the cGAS–STING pathway.

NLRP3 inflammasome is another key inflammatory platform that can be activated by diverse mitochondrial stress signals like mtROS, cardiolipin, and mtDNA [319, 320, 321, 322]. It is significantly in cardiovascular disorder, neurodegenerative disease, osteoarthritis, and other age‐related diseases.

MCC950, a potent and selective small molecule inhibitor of NLRP3, has shown significant effects in a variety of aging models [323]. Studies have shown that in the ovarian aging model, MCC950 can significantly improve ovarian function and fertility, and its effect is equivalent to that of NLRP3 knockout [324, 325]. In another study, MCC950 improved cardiac function and metabolic abnormalities by inhibiting cardiomyocyte pyroptosis in d‐galactose‐induced cardiac aging model [326, 327]. In addition to the highly specific inhibitors such as MCC950, some existing clinical drugs have also been found to have the antiaging potential of inhibiting NLRP3. For example, sodium glucose cotransporter 2 inhibitors may exert immunomodulatory and antioxidant effects by regulating AMPK/SIRT1/PGC‐1α signaling and inhibiting NLRP3 activation [328, 329]; β‐hydroxybutyric acid, the main metabolite in the ketogenic state, has also been confirmed to effectively block the activation of NLRP3 inflammasome, thereby producing anti‐inflammatory effects [330, 331].

Improving mitochondrial health has emerged as an indirect approach to inhibit the activation of NLRP3, which is intricately linked to mitochondrial dysfunction. In the OA model, growth differentiation factor 11 attenuated inflammation and cartilage degradation by blocking NLRP3 inflammasome activation by preventing mitochondrial dysfunction [332]. Similarly, acetyl zingerone, a small molecule compound, was confirmed to inhibit NLRP3 inflammasome activation and chondrocyte pyroptosis by inducing PINK1/Parkin‐mediated mitophagy, eliminating impaired mitochondria, and reducing mtROS production [333]. These studies collectively suggest that enhancing mitophagy and biogenesis is an effective means to control NLRP3‐mediated inflammation.

Of course, cGAS–STING pathway and NLRP3 inflammasome are not two independent units. Studies have found that up regulating the expression of NLRP3 through cGAS–STING pathway can enhance the cell scorch effect mediated by NLRP3 inflammasome [334]. NLRP3, as a negative regulator of cGAS–STING pathway, exists to prevent the over activation of this pathway and avoid causing harmful over inflammatory reactions. NLRP3 defects lead to the abnormal high expression of Type I interferon IFN‐β‐mediated by cGAS–STING after radiation [335].

The cGAS–STING and NLRP3 inflammatory pathways triggered by mitochondrial stress are key links facilitate the aging process and associated diseases. Current research strategies are mainly divided into direct targeting and indirect regulation. Although the preclinical evidence is encouraging, challenges remain in translating these strategies into clinical applications, including tissue targeting of drugs, long‐term safety, and complex network regulation of aging‐related chronic inflammation. Future investigations must elucidate the precise mechanism of these pathways in specific tissues and cell types and explore the therapeutic potential of their inhibitors in human aging and related diseases (Table 3).

**TABLE 3:** Generalized intervention strategies targeting mitochondrial function.

Strategy	Method	Core mechanism	References
Metabolic regulators	Chemical uncoupler	Uncouples OXPHOS, reduces membrane potential, and decreases ROS production at the source	[336]
	UCPs	Mediates proton leak, uncouples respiration, generates heat, and reduces ROS	[337]
Regulators of mitochondrial dynamics	Mdivi‐1	Inhibit excessive division	[338]
	MFN1 agonist (S89)	Small molecules promote MFN1‐mediated mitochondrial fusion	[302]
	MFN2 modulator (MASM7)	Peptidomimetic small molecule that promotes fusion by allosterically activating MFN2	[339]
	OPA1 stabilizer	By inhibiting OPA1 cleavage, increasing active L‐OPA1, stabilizing cristae structures, and improving OXPHOS	[340]
Targeting mitochondria‐derived inflammation	cGAS–STING inhibitor	Inhibit cGAS or STING activity triggered by cytoplasmic mtDNA, blocking the production of Type I interferons and inflammatory factors	[315]
	NLRP3 inflammasome inhibitor	Potently and selectively inhibits the assembly and activation of the NLRP3 inflammasome	[341]
	β‐Hydroxybutyrate (BHB)	The main metabolite in a ketogenic state can block the activation of the NLRP3 inflammasome.	[342]
Mitochondrial biogenesis enhancers	Exercise/calorie restriction	By activating AMPK and SIRT1, and upregulating PGC‐1α, it promotes new mitochondrial biogenesis.	[343]
	AMPK agonist	Simulate exercise, activate AMPK, and downstream activate PGC‐1α	[344]
	Cold exposure	Activate thermogenesis and raise mitochondrial biogenesis through the UCP1 and PGC‐1α pathways	[345]

### 4.2 Disease‐Targeted Intervention

As mentioned above, the broad intervention strategy for mitochondria provides rich options for delaying aging and related diseases. However, aging shows heterogeneity in different tissues and organs, and the disease spectrum caused by aging is also complex and diverse. Therefore, based on the broad strategy, targeted intervention for specific pathological environment and high‐risk organs has become the focus of current translational medicine research. This section will focus on several typical aging‐related diseases—skeletal degenerative diseases, neurodegenerative diseases, CVDs, and metabolic diseases—and review the targeted interventions and their potential at the forefront. Although these diseases affect different tissues and organs, there are similar mitochondrial pathological characteristics behind them. Through the analysis of these disease‐specific pathways, we aim to lay the foundation for the development of precise treatment programs for different clinical backgrounds.

#### 4.2.1 Skeletal Degenerative Diseases

Skeletal degenerative diseases, such as osteoporosis and sarcopenia, are common pathological conditions in the aging process, which seriously affect the quality of life and mobility of the elderly. More and more evidence shows that mitochondrial dysfunction is the core link driving the occurrence and development of these diseases. As a functional whole, the maintenance of the stable state of skeletal muscle and bone depends on the healthy mitochondrial function. In the skeletal system, mitochondrial quality control plays a key role in maintaining the dynamic balance between osteoblasts and osteoclasts. In the muscle system, mitochondria are the basis for maintaining the energy supply, contraction function, and satellite cell regeneration of muscle fibers.

BMSCs have the potential to differentiate into various cell types, including osteoblasts, chondrocytes, and adipocytes [346]. Lineage fate decisions between osteoblasts and adipocytes are mutually exclusive, and age‐related osteoporosis is partly due to the reduced ability of BMSCs from older patients to differentiate into osteoblasts and increased ability to differentiate into adipocytes [149]. And in the pathological environment of osteoporosis, especially in postmenopausal osteoporosis and senile osteoporosis (SOP), BMSCs and osteoblasts show significant mitochondrial dysfunction.

Studies have shown that BMSCs from ovariectomized rats exhibit premature aging, elevated ROS levels, and mitochondrial dysfunction, while the phytoestrogen genistein can rescue the senescence phenotype of cells by targeting estrogen‐related receptor alpha, inducing mitochondrial biogenesis and mitophagy [347]. Similarly, in BMSCs senescence induced by advanced glycation end products and SOP model, it was found that decreased expression of SIRT3 led to impaired mitophagy, while overexpression of SIRT3 could alleviate cellular senescence and bone loss by restoring mitophagy [348, 349]. These findings revealed that enhancing mitochondrial biogenesis and mitophagy by regulating specific targets is a promising strategy to intervene osteoporosis. In addition, the imbalance of mitochondrial dynamics, as well as the dysregulation of mitophagy mediated by PINK1/Parkin and other signaling pathways, have also been confirmed to be involved in the functional disorder of osteoblasts and osteoclasts, and then affect the balance of bone metabolism [122, 350, 351, 352].

In sarcopenia, decreased skeletal muscle mass and functional decline are closely associated with impaired mitochondrial integrity [353]. The abnormal mitochondrial dynamics and decreased efficiency of mitophagy caused by aging cause the accumulation of dysfunctional mitochondria, which in turn trigger oxidative stress, energy crisis, and apoptosis signals, and ultimately damage the function of muscle fibers and satellite cells [354, 355, 356]. A key study revealed that mitochondrial fission is essential for maintaining the regenerative capacity of satellite cells. The blockage of mitochondrial fission caused by aging or gene defects will cause ETC dysregulation, inefficient oxidative phosphorylation metabolism and impaired mitophagy, thus weakening the proliferation and function of satellite cells. While restoring mitochondrial fission or promoting mitophagy can reverse these aging phenotypes [357]. This suggests that targeting mitochondrial dynamics and quality control is one of the fundamental ways to combat sarcopenia.

Based on the above mechanisms, therapeutic strategies targeting mitochondria show great potential. In terms of nutrition and drug intervention, supplementation of nad+ precursor nicotinamide mononucleotide NMN and Trigonelline has been shown to increase intracellular NAD+ levels, enhance mitochondrial function, and improve muscle mass and strength in aging models [357, 358, 359]. The exercise‐induced myofactor Apelin has also been found to reverse age‐related sarcopenia by promoting mitochondrial biogenesis and autophagy [360]. In terms of pharmacological intervention, the use of mitochondrial uncouplers can significantly improve muscle mass and function in aging obesity models by reducing mitochondrial coupling efficiency, activating PINK1–Parkin‐mediated mitophagy, and improving endoplasmic reticulum homeostasis [361]. In addition, emerging studies have also revealed the existence of the gut microbiota bone axis. Specific flora can affect bone metabolism by regulating glutathione synthesis and mitochondrial biogenesis in osteoclasts. Supplementing probiotics is expected to become a new method to prevent osteoporosis [362].

As the hub of cell energy and health, mitochondria play an important role in skeletal and muscle degenerative diseases. Targeting mitochondrial biosynthesis, kinetics, mitochondrial autophagy, and other quality control links, as well as related signal pathways, provides a solid theoretical basis and new treatment scheme for the development of new therapies to prevent and treat osteoporosis and sarcopenia.

#### 4.2.2 Neurodegenerative Diseases

In the complex pathology of neurodegenerative diseases such as AD and PD, mitochondrial dysfunction has been identified as a core and druggable target. Its core mechanisms mainly include the accumulation of damaged mitochondria, blocked mitophagy, oxidative stress, and the resulting neuroinflammation [363, 364, 365]. Together, these processes exacerbate the pathological deposition of Aβ peptide and tau protein in AD, as well as the degeneration of dopaminergic neurons in PD [366, 367]. Based on the in‐depth understanding of these mechanisms, targeting mitochondria, especially enhancing mitochondrial quality control, has become a promising therapeutic field.

A large number of preclinical studies have confirmed that mitophagy can effectively reverse disease‐related pathological changes and cognitive motor deficits. In AD model, using drugs such as urolithin A and NAD^+^ supplements to stimulate mitophagy can not only clear dysfunctional mitochondria through PINK1/Parkin‐dependent pathways, but also significantly reduce the deposition of insoluble Aβ and hyperphosphorylated tau, and reverse memory impairment [366, 368, 369]. It is worth noting that long‐term treatment with urolithin a has also been found to restore lysosomal function, and comprehensively improve the cognition, olfaction and synaptic plasticity of AD model mice by upregulating key molecules such as cathepsin Z, highlighting the importance of repairing the autophagy lysosome pathway [368]. These encouraging preclinical results are gradually translating to the clinic, and some mitophagy inducers have entered the evaluation stage of human clinical trials [370]. At the same time, ApoE4, the most important genetic risk factor for AD, can damage neurons by inducing mitochondrial calcium overload, oxidative stress and kinetic abnormalities. Therefore, in the face of ApoE4 carriers, it is essential to develop specific drugs that can stabilize mitochondrial calcium homeostasis, reduce oxidative damage, or regulate mitochondrial dynamics [371, 372, 373, 374].

In PD, because mutations in PINK1 and parkin genes directly lead to impairment of the mitophagy pathway, this pathway has become one of the most critical therapeutic targets. In addition to exploring gene therapy aimed at restoring PINK1/Parkin function, research has also been devoted to the discovery of noncanonical pathway activators. For example, nifedipine has been shown to directly activate PINK1 independent of Parkin, providing more options for treatment [375, 376]. Enhancing mitophagy helps to timely remove mitochondria damaged by environmental toxins or gene mutations, thus preventing the apoptotic process of dopaminergic neurons [377, 378].

In addition, other emerging therapies targeting mitochondria are also being explored and show great potential. These include mitochondrial transplantation, stem cell‐based approaches, photobiomodulation, and lifestyle interventions through diet and exercise [379, 380, 381, 382]. Given the complexity of the pathology of neurodegenerative diseases, the future treatment paradigm is likely to tend to combination therapy, that is, the simultaneous or sequential application of mitophagy inducers, antioxidants, and anti‐inflammatory drugs, which work synergistically from multiple levels to maximize the protection of neurons and delay or even prevent the progression of the disease.

#### 4.2.3 Cardiovascular Lesions

CVDs, such as HF, myocardial ischemia/reperfusion injury, AS, and vascular aging, are the main causes of death and disability worldwide. In the pathological process of these diseases, mitochondrial dysfunction plays a central role, mainly manifested by ROS overproduction, abnormal energy metabolism, kinetic imbalance, and quality control imbalance [383, 384]. Therefore, targeting mitochondria has become an important direction in the prevention and treatment of CVD.

Mitochondria are the main source of ROS in the cardiovascular system, and excessive ROS can damage endothelial function, promote inflammation, and trigger cell death [385]. MitoQ, a well‐studied compound, can specifically accumulate in the mitochondrial matrix and neutralize ROS. Studies have shown that in aged mice and elderly people, MitoQ treatment can significantly improve vascular endothelial dysfunction and aortic stiffness caused by age or hypertension, and its mechanism is related to reducing mitochondrial oxidative stress, restoring NO bioavailability, and reducing elastic fiber degradation [386, 387, 388]. In hypertension model, MitoQ can also effectively reduce blood pressure and reverse myocardial hypertrophy [389].

Myocardial ischemia/reperfusion injury is a major complication of reperfusion therapy after acute myocardial infarction. Its core link is the abnormal opening of mPTP, which leads to mitochondrial swelling, cytochrome *c* release, and cell necrosis [390, 391]. Therefore, mPTP has become a key intervention target. Cyclosporine A, a classical mPTP inhibitor, exerts protective effects by inhibiting cyclophilin D [392]. In recent years, a novel small molecule cyclophilin inhibitor c105sr was developed, which showed stronger mitochondrial protection and efficacy than cyclosporine A in liver ischemia/reperfusion model [393]. At the same time, natural compounds also showed therapeutic potential, such as curcumin and icariin, which were confirmed to inhibit mPTP opening by upregulating Hes1 and directly acting, respectively, thereby alleviating myocardial and brain ischemia/reperfusion injury [394, 395]. The latest research also revealed the synergistic effect of combined targeting: in the myocardial ischemia/reperfusion model, inhibiting mPTP with cyclosporine A and inhibiting the upstream inflammatory trigger—extracellular RNA with RNase1 can synergistically reduce the infarct size, which provides a strong theoretical basis for the clinical combination of drugs [396].

The continuous fusion and division of mitochondria and selective autophagy are essential for maintaining the energy supply and survival of cardiomyocytes. In HF and ischemia/reperfusion injury, excessive mitochondrial fission is a prominent pathological feature. Studies have shown that adipokine Omentin1 can improve cardiac function, reduce myocardial hypertrophy and infarct size in myocardial ischemia model by upregulating SIRT3/FOXO3a signaling pathway, correcting mitochondrial fusion/division imbalance, and activating PINK1/Parkin‐mediated mitophagy [387].

### 4.3 Emerging Technologies

With the deepening understanding of the core role of mitochondria in aging and related diseases, the frontier of scientific research has shifted from basic mechanism exploration to the development of innovative therapies that can directly intervene and reverse mitochondrial dysfunction. Traditional small molecule drug strategies often have limited efficacy because they are difficult to accurately target‐specific components of mitochondria or overcome heterogeneous barriers. In order to break through these bottlenecks, a series of technologies emerged at the historic moment. They have brought unprecedented hope for the precise targeted treatment of aging‐related mitochondrial diseases from the three dimensions of substitution, repair, and delivery. The fusion and development of these emerging technologies are jointly pushing mitochondrial medicine into a new era of treatment.

#### 4.3.1 Mitochondrial Transplantation and Engineering

Mitochondrial transplantation and engineering represent one of the most cutting‐edge directions in the field of targeted mitochondrial therapy. Its core is to supplement healthy mitochondria to functionally impaired cells through endogenous or exogenous ways to restore their energy metabolism and cell function, which has great therapeutic potential for aging and related diseases characterized by progressive mitochondrial function decline [397, 398].

Traditional mitochondrial transplantation strategies mainly rely on direct transplantation of isolated mitochondria. Preclinical studies have confirmed that direct transplantation of functional mitochondria from healthy tissues into damaged cardiomyocytes, neurons or tendon cells can effectively improve the oxidative phosphorylation capacity of cells, increase ATP production, reduce oxidative stress, and rescue cell apoptosis, thus showing therapeutic potential in age‐related disease models such as myocardial ischemia–reperfusion injury, neurodegenerative diseases, and tendinopathy [399, 400, 401]. However, this technology faces two major obstacles: one is that the isolated mitochondria have poor stability and rapid loss of activity in the in vitro environment; The second is the lack of targeting to diseased cells, which limits its therapeutic efficiency and clinical application [402].

In order to overcome these obstacles, researchers turned to biomimetic and engineering strategies. Extracellular vesicle based mitochondrial delivery is an important breakthrough. Cells can naturally encapsulate mitochondria in extracellular vesicles to form mitochondrial vesicles. These vesicles not only provide a natural protective barrier for mitochondria to maintain their activity and functional stability, but also endow them with natural targeting ability through molecules on their membrane surface, enabling them to be preferentially taken up by damaged cells [403, 404]. Among them, mesenchymal stromal cells are an ideal source for the generation of such vesicles, and their mediated mitochondrial transfer has shown repair effects in models of neurological, vascular, and pulmonary diseases [403, 405, 406].

Recent studies have further created “super donor cells” by engineering means to significantly improve the yield and quality of mitochondrial vesicles. A pioneering work found that upregulation of CD38 expression in mesenchymal stem cells by nonviral gene vectors could activate the CD38/IP3R/Ca^2+^ signaling pathway, significantly promoting mitochondrial Ca^2+^ levels and extracellular vesicle mitochondria release [407]. The engineered “super mitochondrial vesicles” not only have three times more mitochondria than the control group, but also have stronger mitochondrial function and better stability in vivo and in vitro. In the classic mitochondrial disease model of Leber hereditary optic neuropathy, such engineered vesicles can effectively repair mtDNA defects, restore energy metabolism of retinal cells, and significantly delay vision loss, providing a powerful new tool for the treatment of aging‐related mitochondrial dysfunction diseases.

In addition, nanotechnology‐assisted mitochondrial delivery also provides innovative solutions to solve the problems of targeting and stability. For example, researchers have developed a strategy to modify NO releasing nanomotors on the surface of mitochondria, and constructed chemically oriented “nanomotor mitochondria” that can actively target ischemic myocardial tissue. Delivered by oral enteric coated capsules, this engineered mitochondria can cross the gastrointestinal barrier and enter the circulatory system, and are highly enriched in the injured myocardium [408]. By regulating the transcriptional program of cardiac metabolism, it successfully delays the progression of ischemic heart disease [409]. This noninvasive delivery method has brought new hope for improving the prevalent cardiovascular function decline in aging individuals.

The field of mitochondrial transplantation is evolving from simple organ body supplement to intelligent and targeted delivery system based on engineering technology. By combining extracellular vesicle biology, genetic engineering, and nanotechnology, a new generation of mitochondrial transplantation strategy is expected to reverse mitochondrial dysfunction in aging and related diseases more efficiently and safely, with broad prospects for clinical transformation.

However, mitochondrial replacement technologies (MRT) derived therefrom refer to a series of technologies that transfer nuclear DNA from oocytes or fertilized eggs containing defective mtDNA to enucleated oocytes or fertilized eggs with normal mitochondria through micromanipulation [410]. This technology is intended to help mothers with severe mitochondrial disease due to carrying defective mtDNA to produce healthy offspring with their own genetic material and blood relationship. As a means to prevent severe maternal mitochondrial genetic diseases, the safety and effectiveness of MRT still need more scientific data to support. Academic circles have long debated MRT‐related scientific, ethical, social, and legal issues. The technical risk of MRT is related to the residual mtDNA of patients, the increase of copy number, and the incompatibility between patient DNA and donor mtDNA. In terms of ethical values, the genetic information of MRT offspring comes from three people, which will impact the inherent family view. At the same time, the potential application of this technology is still accompanied by ethical and social disputes, such as “three parent babies,” genetic modification of human reproductive system, and so on. Different countries have different ethical attitudes and regulatory measures toward such issues, which pose major challenges to the development and governance of MRT.

#### 4.3.2 Gene Editing and Gene Therapy​

Mutation accumulation of mtDNA is a key factor driving cellular senescence and a variety of age‐related diseases. However, due to the existence of mitochondrial membrane and unique physiological environment, direct editing of mtDNA was once a great challenge. In recent years, the emergence of a series of breakthrough technologies has provided unprecedented precision tools for correcting mtDNA defects at the source and treating aging‐related mitochondrial diseases.

The CRISPR‐derived base editing tool targeting mtDNA is a revolutionary progress in this field. Among them, the cytosine base editor (DdCBE) derived from the bacterial toxin DddA, mitochondrial targeted zinc finger deaminases, and TALE linked deaminases (TALED) are the most attractive [411, 412, 413]. DdCBE can realize the conversion from C • G to T • A of specific sites on mtDNA, while TALED has further broken through the technical barriers and realized the conversion from A • T to G • C. Together, they realize the theoretical coverage of all transition mutations [413, 414]. These tools do not produce DNA double strand breaks, nor depend on the replication state of mtDNA, so they are more safe and efficient. Although the original TALED has significant RNA targeting effect and cytotoxicity, the substrate binding site of its deaminase TadA8e has been modified through protein engineering, and a high fidelity variant has been successfully developed, which can reduce RNA targeting editing by more than 99%, and eliminate the toxicity to mouse embryo development, laying the foundation for the generation of disease models and safe treatment of Leigh syndrome [415].

Subsequent research has further improved editing efficiency and reduced nuclear genome miss through strategies such as fusing highly active DddA variants and optimizing nuclear output signals, which has promoted such tools to move toward clinical application [416, 417]. The latest research reveals that the action mechanism of TALED depends on the way of base excision and repair, and accordingly, an enhanced eTALED with higher editing efficiency and less bystander editing has been developed [416]. This new strategy to improve editing efficiency and accuracy by controlling and optimizing the internal repair mechanism of cells has laid a solid foundation for the generation of high fidelity disease models and safe future gene therapy, highlighting the rapid development of this field from tool discovery to mechanism understanding, and then to performance optimization.

The regulation of mtDNA heterogeneity and mitochondrial targeted transcription activator like effector nucleases (mitoTALENs) are another important therapeutic pathway. Many pathogenic mtDNA mutations exist in a “heterogeneous” state, that is, cells contain both wild‐type and mutant mtDNA. When the proportion of mutant mtDNA exceeds a specific threshold, it will cause disease [418]. Therefore, it has become an attractive therapeutic strategy to specifically eliminate mutant mtDNA to transform the proportion of heterogeneity below the pathological threshold [419]. Customized mitoTALENs can accurately identify and combine specific mutant mtDNA sequences and introduce double strand breaks, so as to selectively eliminate mutant genomes by using the degradation mechanism of mitochondria itself, enabling the dominant proliferation of wild mtDNA [420]. This strategy has been successfully verified in cell models and animal models and can reduce the proportion of heterogeneity to the therapeutic level, bringing hope for the treatment of maternal inherited mitochondrial disease caused by point mutations.

At the same time, adeno‐associated virus (AAV)‐mediated gene therapy plays a key role in compensating for functional defects of mitochondrial‐related genes in the nuclear genome, which can also lead to secondary mitochondrial dysfunction, and is common in the aging process [421]. Although it is difficult for AAV to effectively target mitochondrial matrix as a direct delivery editing tool, its safety and efficiency in delivering nuclear genes make it an ideal vector for the treatment of nuclear gene mitochondrial communication disorders [422]. For example, in the study of long‐chain 3‐hydroxyacyl coenzyme A dehydrogenase deficiency, a fatty acid oxidation disorder that leads to progressive retinitis pigmentosa, researchers successfully delivered the wild‐type HADHA gene to retinal pigment epithelial cells derived from patients with induced pluripotent stem cells using recombinant AAV, effectively saving cell lipid peroxidation stress and mitochondrial functional defects [423]. In addition, in order to overcome the problem of low transfection efficiency of AAV in chronic inflammatory tissues such as aging or diabetic wounds, the emerging bionic strategy wraps AAV in extracellular vesicles to form EV‐AAV. This composite system not only improves the stability and cell uptake efficiency of AAV, but also combines with hydrogel and other sustained‐release systems to achieve long‐term and controllable transgene expression in local areas, while regulating the inflammatory microenvironment, providing a multifunctional platform for the treatment of age‐related tissue repair disorders [424].

#### 4.3.3 Nanotechnology Targeting Mitochondria and Drug Delivery Systems

As the energy factory and apoptosis regulation center of cells, mitochondria dysfunction is one of the core links of aging and related diseases. However, due to the unique double membrane structure and highly negative membrane potential of mitochondria, it is a great challenge to deliver therapeutic drugs to the mitochondrial matrix efficiently and specifically [425]. The emerging nanotechnology and intelligent drug delivery system provide a promising strategy to solve this problem and lay a foundation for the realization of precision medicine.

At present, the nano delivery system targeting mitochondria mainly relies on the active targeting strategy, that is, by modifying the mitochondrial oriented molecules on the mitochondrial carrier. Among them, cationic amphiphilic molecules such as TPP^+^ are most widely used [426, 427]. TPP^+^ can use the high negative potential inside the mitochondrial membrane to drive the nano carrier through the mitochondrial membrane through electrostatic effect [428]. Studies have shown that TPP^+^ modification on the surface of lipid nanoparticles (LNPs), polymers, or inorganic nanoparticles can significantly enhance their enrichment in mitochondria [429, 430]. For example, in the model of acute renal injury, the TPP^+^‐modified cerium oxide nanoparticles loaded with atorvastatin can respond to the high ROS environment at the focus and release drugs. At the same time, TPP^+^ guides the nanoparticles to target mitochondria to eliminate excessive ROS, effectively alleviating mitochondrial oxidative stress and apoptosis, and demonstrating its therapeutic potential in aging‐related oxidative damage diseases [431]. Similarly, in the psoriasis model, TPP‐modified ceria nanoparticles were coloaded with all trans retinoic acid in flexible nanoliposomes, which were successfully targeted to the mitochondria of the diseased skin after percutaneous delivery, scavenging ROS and improving the symptoms of filariasis through its valence switching ability [432].

Among many nanocarriers, LNPs are favored due to their good biocompatibility, loading diversity and easy functionalization [433]. In addition to being small molecule drug carriers, LNPs have shown great potential in nucleic acid drug delivery [434]. For example, in the myocardial ischemia/reperfusion injury model, researchers constructed LNPs loaded with long noncoding RNA Oip5–as1 and modified cardiomyocyte targeting peptides. The system successfully delivered Oip5–as1 to cardiomyocytes and improved cardiac function by inhibiting p53 pathway to protect mitochondrial function and reduce apoptosis, which provides a new idea for the treatment of aging‐related cardiac function decline [435].

On the other hand, the MITO‐Porter system developed by the team of Japanese scholars Yamada and Harashima represents an innovative membrane fusion delivery mechanism [436, 437]. MITO‐Porter is a liposomal carrier whose membrane material is specially designed to fuse with the mitochondrial membrane to deliver macromolecules directly into the mitochondrial matrix [436]. Studies have confirmed that the use of Mito Porter successfully delivered CoQ10 to macrophage mitochondria, significantly enhanced its antioxidant effect and improved mitochondrial respiratory function, which provided a new method for regulating immune metabolism to intervene aging‐related chronic inflammation [438]More importantly, MITO‐Porter realized the proof of concept of mitochondrial gene therapy. By delivering antisense oligonucleotides targeting mitochondrial encoded mRNAs to the mitochondrial matrix, the knockdown of specific mRNAs was successfully achieved, which in turn affected MMP and ATP production [439]. This opens up a new therapeutic approach to correct the accumulated mtDNA mutations and functional defects in senescent cells.

**FIGURE 3:**
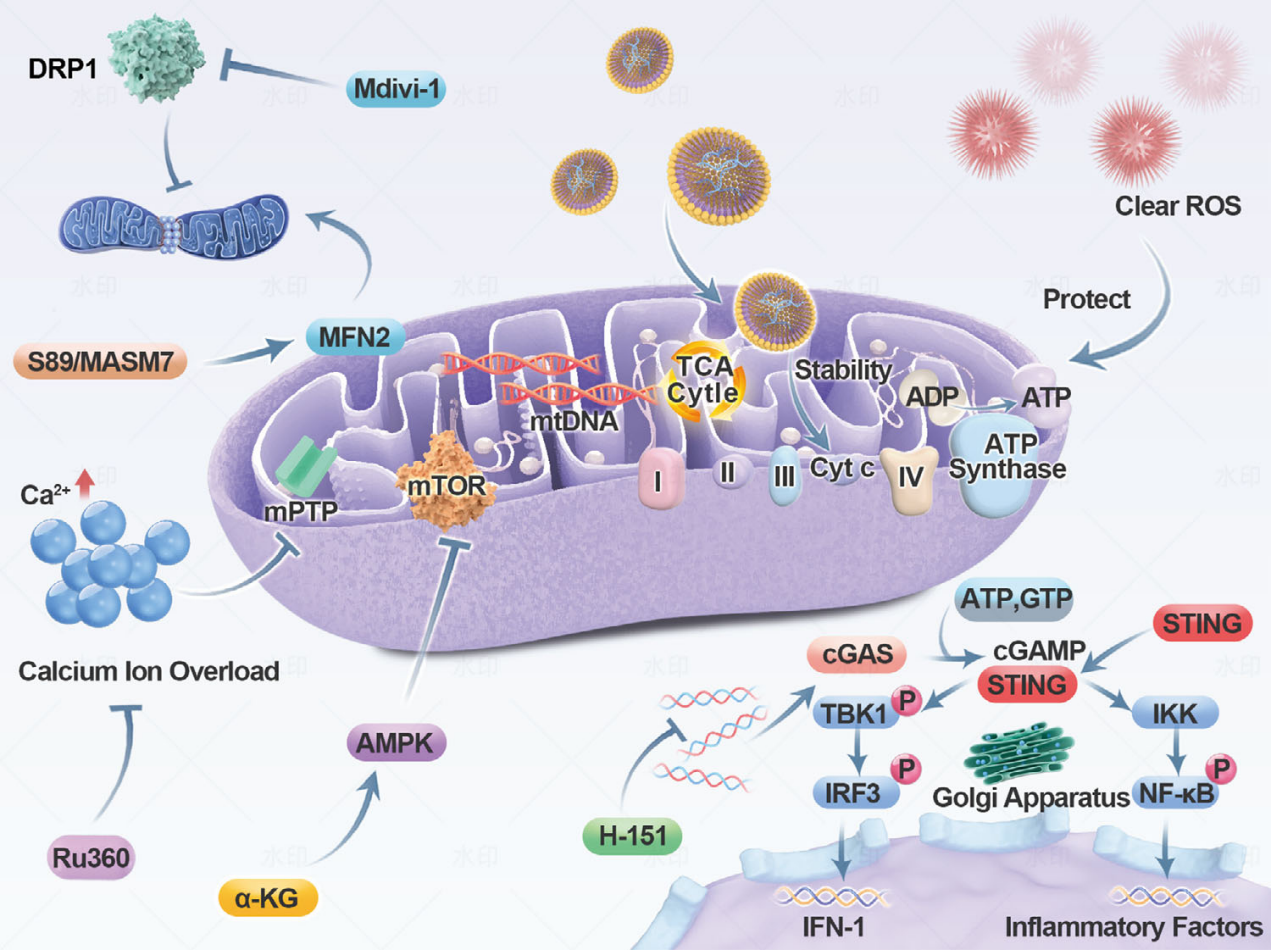
Target distribution of mitochondrial targeted therapy strategy. The targets of mitochondrial targeted therapy are mainly distributed in the outer membrane, inner membrane, and matrix of mitochondria. They are respectively involved in kinetic inhibitor Mdivi‐1, inflammatory inhibitor H‐151, calcium regulator Ru360, and metabolic regulator α‐KG; a variety of antioxidants for mitochondrial therapy mainly play a role in protecting ETC by clearing ROS. Mdivi‐1 inhibits DRP1 translocation, thereby blocking mitochondrial cleavage; S89/MASM7 promotes mitochondrial outer membrane fusion by activating MFN2; Ru360 plays a role by inhibiting calcium overload and blocking the opening of mPTP in the intima. α‐KG can activate AMPK, inhibit mTOR, restore TCA flux, and improve epigenetics; DddA‐derived cytosine base editor (DdCBE) modifies mtDNA point mutations to restore ETC function. At the same time, inflammatory inhibitor H‐151 inhibits inflammation by inhibiting the cGAS–STING pathway.

Despite its promising prospects, mitochondria targeted nanotechnology still faces many challenges. First, the delivery journey of nanocarriers from extracellular to mitochondria needs to overcome multiple physiological barriers, including cellular uptake, endosomal escape, and ultimately precise mitochondrial recognition and intake. Second, the safety of the carrier itself needs close attention. For example, some studies have pointed out that mRNA‐LNPs of some components may activate the NLRP3 inflammasome by causing lysosomal rupture and mitochondrial ROS production, which may have complex effects on the basis of chronic low‐grade inflammation already existing in aging [440]. Future research directions will focus on developing more intelligent stimuli responsive vectors to improve specificity, combine multiple targeting strategies, and explore its therapeutic potential and long‐term biosafety in more aging‐related disease models (Figure 3).

### 4.4 Translational Advances: From Preclinical Models to Clinical Trials

More and more preclinical evidence indicates the therapeutic potential of mitochondrial targeted intervention. Although many strategies are still limited to animal models, some strategies have now entered clinical trials, providing key insights into their safety, tolerance and efficacy in humans. This section focuses on the representative transformation progress of some treatment strategies, with detailed information comprehensively summarized in Table 4.

**TABLE 4:** Summary of key preclinical and clinical studies in mitochondria‐targeted therapies.

Strategy	Compound	NCT no.	Phase/status	Primary objectives	Preliminary findings
Mitochondria‐targeted antioxidants	MitoQ	NCT00329056	Phase II/completed	Parkinson's disease	Well‐tolerated; did not slow disease progression
		NCT02364648	Phase IV/completed	Chronic kidney disease	Improved macrovascular endothelial function, arterial hemodynamics, and microvascular function; well tolerated
	SkQ1	NCT03764735	Phase II/completed	Dry eye syndrome	Significantly improved corneal functional state and tear film stability, reduced corneal damage
	Elamipretide (SS‐31, MTP‐131)	NCT03323749	Phase III/completed	Primary mitochondrial myopathy	Did not meet primary endpoint in overall population; post hoc analysis showed significant benefit in mtDNA replisome disorder subgroup
		NCT06373731	Phase III/recruiting	Dry age‐related macular degeneration	50% enrollment reached; evaluating efficacy, safety, and pharmacokinetics of daily SC injections
NAD^+^ precursors	NMN	NCT04273165	Phase I/II/completed	Healthy aging	Well‐tolerated; increased NAD^+^ levels
Mitochondrial transplantation	Autologous mitochondrial transplantation	NCT02851758	Early phase I/recruiting	Myocardial ischemia–reperfusion injury	Pilot study in pediatric patients: associated with successful separation from ECMO and enhanced ventricular strain

Data sources—clinical registration website.

ECMO: extra corporeal membrane oxygenation.

Mitochondria‐targeted antioxidants represent the most clinically advanced class of mitochondrial therapeutics. MitoQ, which conjugates ubiquinone to a TPP^+^ cation, has been evaluated in over 25 human clinical trials spanning multiple disease indications. The Phase II PROTECT trial (NCT00329056) evaluated MitoQ as a disease‐modifying therapy in newly diagnosed PD patients. Although MitoQ failed to slow motor progression over 12 months, the trial established an excellent long‐term safety and tolerability profile in a neurodegenerative population [217]. More recently, a Phase IV study in chronic kidney disease patients (NCT02364648) demonstrated that MitoQ improved macrovascular endothelial function, arterial hemodynamics, and microvascular function, further supporting its multiorgan therapeutic applicability [441]. SkQ1, another TPP^+^‐conjugated antioxidant containing plastoquinone, has achieved regulatory approval in Russia for the treatment of dry eye disease, representing the first mitochondria targeted antioxidant to reach the market [220]. In preclinical models of accelerated aging, SkQ1 extended lifespan, preserved mitochondrial ultrastructure, and delayed age‐related functional decline [218]. Clinical trials for other antiaging indications are currently underway, including the application of neurodegenerative diseases and CVDs.

Elamipretide, a tetrapeptide that selectively binds to cardiolipin on the inner mitochondrial membrane, has demonstrated protective effects across a wide range of preclinical models, including aging‐related diastolic dysfunction, ischemia–reperfusion injury, and acute kidney injury [24, 221]. The Phase III MMPOWER‐3 trial (NCT03323749) evaluated the role of elamipretide in patients with primary mitochondrial myopathy. Although the trial did not meet the main goal of the overall population, prespecified post hoc analyses revealed a significant clinical benefit in the mtDNA replisome disease subgroup, suggesting that treatment response may depend on the specific genetic etiology [442]. It is also currently being evaluated in a Phase III trial for dry age‐related macular degeneration (NCT06373731), highlighting the expanding clinical footprint of this cardiolipin‐targeted peptide.

Although most emerging technologies are still in preclinical or early clinical development stage, their rapid progress and transformation potential deserve our special attention. Mitochondrial transplantation has been applied in a limited number of clinical case reports, mainly for pediatric cardiogenic shock after ischemia–reperfusion injury. In a preliminary study (NCT02851758), autologous mitochondria were directly injected into the injured myocardium of pediatric patients requiring extracorporeal membrane oxygenation (ECMO), a process associated with successful separation of ECMO and enhanced ventricular strain [443]. Despite these encouraging advances, a critical gap remains between preclinical success and clinical trial outcomes. Species‐specific differences in mitochondrial biology, the heterogeneity of mitochondrial diseases, the lack of sensitive and effective biomarkers of mitochondrial function for patient stratification and endpoint assessment, and the difficulty in reproducing the complex nature of human aging in animal models are all challenges for this therapy.

## 5 Key Obstacles in Mitochondrial‐Targeted Therapies

### 5.1 Heterogeneity of Mitochondria

Targeting mitochondria as a strategy to treat aging‐related diseases, the primary obstacle is the significant heterogeneity of mitochondria. This heterogeneity is not random noise, but highly ordered biological characteristics embodied in tissues, time, individuals, and cells, which profoundly affect the clinical manifestations, progress, and response to treatment strategies of the disease.

#### 5.1.1 Tissue‐Specific Differences

Mitochondria show profound specificity in different tissues, which is not only reflected in their morphology and quantity, but also the fundamental differences in their functions and metabolic preferences. This tissue specificity directly determines the susceptibility of different organs to mitochondrial dysfunction and their performance during aging.

Mitochondria of continuously working tissues such as myocardium and skeletal muscle optimize oxidative phosphorylation efficiency to maintain high‐intensity ATP production [444, 445, 446, 447]. On the contrary, neuronal mitochondria need to be distributed along long axons and dendrites and are extremely sensitive to calcium buffering and oxidative stress, which explains why specific regions of the brain are particularly vulnerable in neurodegenerative diseases such as PD [448]. Liver mitochondria undertake unique functions such as β‐oxidation and detoxification, and their metabolic pathways are quite different from those of nerve or muscle tissues [449, 450].

Large‐scale sequencing studies of a variety of human tissues have found that loci and alleles with high‐frequency heterogeneity are highly specific in different tissues [451]. For example, a large amount of nonsynonymous heterogeneous accumulation associated with functional impairment was observed in the liver, which was interpreted as a positive selection of “the slowest survival,” aiming to reduce the harmful by‐products produced by liver metabolism [452].

This tissue specificity means that a drug or treatment that enhances mitochondrial biogenesis or respiratory function may bring benefits to muscle, but cause unforeseen consequences in the liver or brain with different metabolic set points. Therefore, a successful mitochondrial targeted therapy must consider the accumulation of drugs in a specific tissue and its impact on the unique mitochondrial physiology of that tissue.

#### 5.1.2 Stage‐Dependent Functional Changes

The function and quality of mitochondria are not static, but dynamic evolution with individual aging and disease progression. Therapeutic strategies must match the mitochondrial pathophysiological characteristics at different stages of the disease, which means that effective interventions at one stage may be ineffective or even harmful at another stage.

In the early stages of aging or disease, mitochondrial dysfunction manifests as mild oxidative stress and decreased energy output [453]. Studies have shown that in the early stage of aging mouse brain, the increase in heterogeneity of mitochondrial size is highly correlated with the decline of Complex I‐related respiratory function, suggesting that the early decline of mitochondrial quality control mechanism is an initial event of aging [454]. Over time, problems such as mtDNA mutations, disordered protein import, and impaired mitophagy gradually accumulate [91, 131, 455, 456]. For example, in the process of cellular senescence, mitochondrial network fragmentation will lead to significant random uneven distribution of nuclear encoded mitochondrial proteins among individual mitochondrial fragments, and this proteome heterogeneity directly exacerbates the decline of mitochondrial function [457]. Finally, in the late stage of the disease, irreversible loss of mitochondrial mass and sustained activation of mitochondrial‐mediated apoptosis pathway may occur [458]. Studies have shown that in senescent hematopoietic stem cells, the MMP can be used as a distinguishing marker between functionally young and senescent cells, and enhancing the membrane potential by pharmacological means can reverse the aging‐related decline in function and the transcriptional program of myeloid differentiation preference [451]. This suggests that interventions targeting specific functional stages may reshape the physiological age of cells.

#### 5.1.3 Interindividual and Cell‐to‐Cell Heterogeneity

**FIGURE 4:**
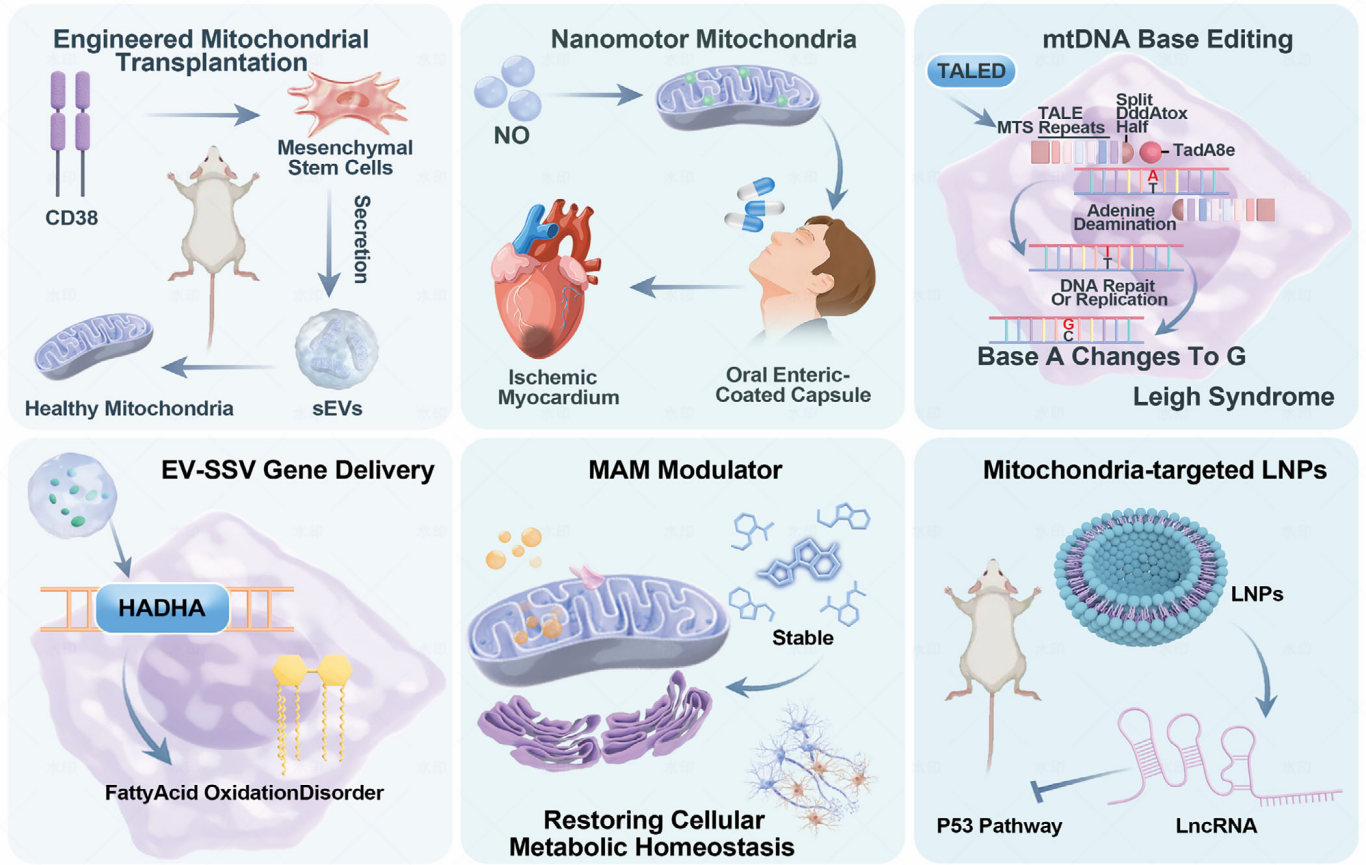
New technologies for mitochondria targeting. The emerging technologies targeting mitochondria mainly include engineered mitochondrial transplantation, oral nano motor mitochondria, mtDNA base editing, EV‐AAV gene delivery, MAM regulators, and mitochondrial targeting LNPs. These new therapies have achieved substantial therapeutic effects in the mouse model of Leber hereditary optic neuropathy, Leigh syndrome, ischemic heart disease rat model, long‐chain 3‐hydroxyacyl coenzyme A dehydrogenase deficiency (LCHADD), and myocardial ischemia injury mouse model.

Mitochondrial heterogeneity also exists among individuals and even among cells in the same tissue. This heterogeneity at the micro level is the main challenge and basis for realizing personalized medicine. The genetic background of an individual, especially the level of mtDNA haplotype and heterogeneity, is a key factor in determining disease susceptibility and clinical manifestations [459]. Proteomic studies of cerebrospinal fluid in PD revealed distinct molecular pathological pathways in different genetic backgrounds, including neuroinflammation, mitochondrial dysfunction, and lysosomal degradation abnormalities [448, 460, 461]. This proves that even if the same disease is diagnosed, there are huge individual differences in the mitochondrial‐related molecular mechanism behind it (Figure 4).

Single cell technology revealed that even in the same tissue, mitochondria of adjacent cells may be in different health and mutation load states. Single cell mtDNA sequencing revealed that there were low‐frequency mtDNA mutations in aging mouse tissues, and cells carrying high variant allele frequencies were more common in aging tissues [462]. More importantly, the selective elimination of nonsynonymous mtDNA mutations in the population does not originate from simple random drift, but is dominated by selection at the cellular fitness level; It is worth noting that the “direction” of this selective effect—that is, whether a specific mtDNA mutation is harmful, neutral, or beneficial—depends entirely on the microenvironment in which the cell is located. For example, under the specific pressure of oligomycin, functionally defective Complex I mutations can instead be positively selected and accumulated. This discovery fundamentally updates our understanding of the mechanism of mtDNA in aging and disease: the accumulation of age‐related mtDNA mutations may not be the passive result of functional decline, but may also be the active selection of cells to adapt to survival in a specific environment. This provides a key theoretical cornerstone for the development of individualized therapies targeting mitochondria, suggesting that effective treatment strategies must fully consider the specific genetic background and pathophysiological environment of patients [461].

The above multilevel heterogeneity together constitutes the fundamental obstacle of “one size fits all” mitochondrial therapy. The future treatment paradigm must turn to personalized medicine, which relies on precise diagnosis and precise intervention. This requires drawing an individual's “mitochondrial map” by integrating multi omics information, and according to this, developing innovative therapies that can specifically correct specific mutations, remodel specific functional defects, or clear specific harmful cell populations, so as to ultimately overcome the obstacles brought by heterogeneity and achieve effective treatment of antiaging‐related diseases [463].

### 5.2 Clinical Translation Issues

Although preclinical studies have demonstrated the great potential of targeting mitochondria in the treatment of aging‐related diseases, its successful translation to the clinic still faces a series of severe challenges. Drug delivery efficiency and targeting specificity are the primary obstacles. As an intracellular organelle with double membrane structure, the precise targeting of mitochondria needs to overcome the multiple barriers of cell membrane and mitochondrial membrane. Although nanocarriers modified with mitochondrial targeting ligands such as TPP have been widely developed and have shown good targeting and therapeutic effects in disease models such as acute kidney injury, the in vivo biodistribution, tissue penetration efficiency and off target effects of these systems still need to be optimized [431, 464, 465; 466]. For example, the size, surface charge, and morphological properties of nanoparticles profoundly affect their ability to clear the kidney, enrich tumors, or cross the blood–brain barrier. However, the behavior of most delivery systems in the complex human physiological environment and aging microenvironment is still not fully understood.

Second, pharmacokinetics and biosafety are the core concerns of clinical translation. Many mitochondrial drugs that are effective in vitro or animal models face problems of low bioavailability, rapid metabolism, or nonspecific distribution in humans. For therapies aimed at long‐term intervention in the aging process, its long‐term biocompatibility and potential cumulative toxicity are the focus of regulators and clinicians. For example, although some metal nanoparticles have excellent ROS scavenging ability, their long‐term retention in the body may trigger immunogenic reactions or unpredictable organ toxicity [467]. Therefore, the development of new, biodegradable delivery systems such as fully biocompatible tantalum antioxidant NanoShield to achieve effective clearance after treatment is an important direction to promote its clinical application [466].

At the same time, the complexity of disease models and the difficulty in selecting clinical endpoints also significantly hindered the transformation process. Many aging‐related diseases are chronic, progressive and highly heterogeneous. The scientific statement of the European Society of Cardiology clearly pointed out that in the field of myocardial ischemia, although a variety of mitochondrial protection strategies in preclinical studies had significant effects, they failed to achieve success in clinical trials. This is partly due to the inability of animal models to fully simulate the complex pathophysiological background of human diseases, including chronic diseases, the combination of multiple drugs, and the impact of aging itself on mitochondrial function [468]. In addition, how to choose a sensitive and specific treatment rather than long‐term dependence is also a major challenge in clinical trial design.

Individual heterogeneity and treatment time are the difficulties in implementing precision medicine. As mentioned earlier, there is great heterogeneity in mitochondria between individuals and different tissues, which means that a therapy may only be effective for a subset of patients with a specific genetic background or disease stage. For example, gene therapy for mitochondrial diseases caused by mtDNA mutations, although conceptually promising, faces obstacles in practical applications such as carrier delivery efficiency, immunogenicity, and how to balance the level of heterogeneity between tissues [469]. At the same time, for acute injury or chronic degenerative lesions, the timing and course of intervention are crucial. Premature or too late, too short or too long treatment may not produce benefits or even be harmful.

## 6 Future Perspectives and Conclusion

Mitochondria, as the energy and signal center of cells, its dysfunction has become the core driving force of aging and a series of aging‐related diseases. From the instability of mtDNA and ROS imbalance, to the disruption of calcium homeostasis, metabolic abnormalities, and finally trigger systemic inflammatory aging, the multidimensional roles of mitochondria in the aging process constitute a complex network. This review systematically expounds the decline of mitochondrial quality control system during aging, and how these changes are embodied in musculoskeletal, neurological, cardiovascular, and metabolic diseases. Although therapeutic strategies targeting mitochondria, from classic antioxidants to emerging gene editing and nanotechnology, have shown great potential, we still face a series of key scientific and clinical challenges.

Mitochondrial dysfunction is an early event in many aging‐related diseases, but its progression is slow. Therefore, in clinical trials, in addition to traditional disease‐specific endpoints, biomarkers that can sensitively reflect mitochondrial function should be included, such as circulating cell free mtDNA, specific metabolite profiles, or mitochondrial respiratory function indicators in muscle tissue. In addition, population stratification according to the mitochondrial function phenotype of patients will be more likely to identify the real benefit groups in clinical trials.

## Author Contributions

Zijie Xiang: data curation, conceptualization, investigation, and writing–original–draft. Yu Chen: data curation, conceptualization, investigation, and writing–original–draft. Xishui Liu: data curation, investigation, and writing–original–draft. Haowen Lu: conceptualization, investigation, and writing–original–draft. Yuqing Yang: conceptualization, investigation, and writing–original–draft. Lei Xing: data curation and revising the manuscript. Yu Zhang: data curation and revising the manuscript. Chuandong Lang: revising the manuscript. Siming Zhang: revising the manuscript. Shixiang Zhao: revising the manuscript. Youzhi Hong: supervision, project administration, and revising the manuscript. Jiaxiang Bai: supervision, project administration, and revising the manuscript. Yusen Qiao: conceptualization, supervision, project administration, and revising the manuscript. All authors have read and approved the final manuscript.

## Funding

This review work was supported by the National Natural Science Foundation of China (No. 82402780, No. 82572432, No. 82573230) and the Scientific Research Project of Anhui Provincial Health Commission (No. AHWJ2024Aa20475).

## Conflicts of Interest

The authors declare no conflicts of interest.

## Ethics Statement

The authors have nothing to report.

## Data Availability

The authors have nothing to report.
